# Inhibition of inflammasome activation by *Coxiella burnetii* type IV secretion system effector IcaA

**DOI:** 10.1038/ncomms10205

**Published:** 2015-12-21

**Authors:** Larissa D. Cunha, Juliana M. Ribeiro, Talita D. Fernandes, Liliana M. Massis, Chen Ai Khoo, Jennifer H. Moffatt, Hayley J. Newton, Craig R. Roy, Dario S. Zamboni

**Affiliations:** 1 Department of Cell Biology, Medical School of Ribeirão Preto, University of São Paulo (FMRP/USP), Ribeirão Preto, Sao Paulo 14049-900, Brazil; 2 Department of Microbiology and Immunology, University of Melbourne at the Peter Doherty Institute for Infection and Immunity, Melbourne, Victoria 3000, Australia; 3 Department of Microbial Pathogenesis, Yale University School of Medicine, New Haven, Connecticut 06536, USA

## Abstract

*Coxiella burnetii* is a highly infectious bacterium that promotes its own replication in macrophages by inhibiting several host cell responses. Here, we show that *C. burnetii* inhibits caspase-1 activation in primary mouse macrophages. By using co-infection experiments, we determine that the infection of macrophages with *C. burnetii* inhibits the caspase-11-mediated non-canonical activation of the NLRP3 inflammasome induced by subsequent infection with *Escherichia coli* or *Legionella pneumophila*. Genetic screening using flagellin mutants of *L. pneumophila* as a surrogate host, reveals a novel *C. burnetii* gene (IcaA) involved in the inhibition of caspase activation. Expression of IcaA in *L. pneumophila* inhibited the caspase-11 activation in macrophages. Moreover, *icaA*^*-*^ mutants of *C. burnetii* failed to suppress the caspase-11-mediated inflammasome activation induced by *L. pneumophila*. Our data reveal IcaA as a novel *C. burnetii* effector protein that is secreted by the Dot/Icm type IV secretion system and interferes with the caspase-11-induced, non-canonical activation of the inflammasome.

## Abstract (web summary)

*Coxiella burnetti* primarily infects alveolar macrophages and causes an acute form of pneumonia called Q fever. Cunha *et al*. describe a type IV secretion effector, termed IcaA, expressed by *Coxiella burnetii* that inhibits inflammasome activation and therefore may contribute to innate immune evasion by bacteria.

## Graphical abstract

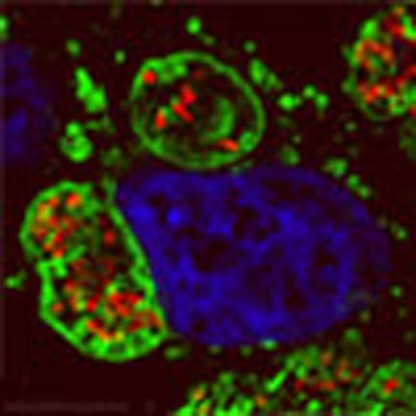

Innate immune cells have developed systems to signal and mount appropriate responses to pathogenic microbes by recognizing conserved molecules present in microbes (pathogen-associated molecular patterns, PAMPs)^1^. For intracellular pathogens, cytosolic nucleotide binding domain and leucine rich repeat containing proteins (NLR) play a pivotal role in recognizing and mounting effective immune responses. Caspase-1 activation mediated by NLR leads to interleukin-1β (IL-1β), IL-18, IL-1α release, membrane permeabilization and a specific form of inflammatory cell death called pyroptosis^2^. The activation of caspase-1 by NLR occurs in a molecular platform known as an inflammasome^3^. Notably, the adaptor protein apoptosis-associated speck-like protein containing a carboxy-terminal CARD (ASC) is involved in the structure of different inflammasomes^2^. Several NLR, including NLRC4, NLRP1, NLRP3, NLRP6, NLRP12, neuronal apoptosis inhibitory protein 2 (NAIP2), NAIP5, NAIP6 and the cytosolic non-NLR receptor absent in melanoma 2 (AIM2), have been shown to activate the inflammasome in response to molecules derived from the different classes of pathogens. In particular, the specialized secretion systems of pathogenic Gram-negative bacteria have been shown to be a direct PAMP for inflammasome activation, and to be involved in processes that lead to delivery of specific PAMPs into the host cell cytoplasm^4^,^5^,^6^,^7^,^8^. In the case of the intracellular Gram-negative bacterium *Legionella pneumophila*, caspase-1 activation is readily triggered in response to the recognition of bacterial flagellin by NAIP5 and NLRC4, a process that requires the expression of a functional type IV secretion system (T4SS) called defect in organelle trafficking/intracellular multiplication (Dot/Icm)^9^,^10^,^11^. Most recently, caspase-11 has emerged as an important regulator of inflammasome activation in response to the lipopolysaccharide (LPS) of Gram-negative bacterial species that either express a secretion system or escape to the host cell cytosol^12^,^13^,^14^,^15^,^16^,^17^,^18^. Caspase-11 mediates NLRP3-dependent caspase-1 activation (called the non-canonical activation of the NLRP3 inflammasome), but also induces a caspase-1-independent membrane permeabilization that leads to pyroptosis and the release of inflammatory cytokines, including IL-1α.

Given the presence of a large repertoire of intracellular pattern-recognition receptors mediating inflammasome activation and pyroptotic cell death, the bona fide intracellular pathogens have developed efficient strategies to manipulate host cell functions and subvert host surveillance (reviewed in Cunha *et al.*^19^). In this sense, the expression of specialized secretion systems that mediate the injection of pathogen effector proteins directly into host cell cytosol constitutes a widespread strategy used by intracellular bacterial pathogens to manipulate host cell functions^20^,^21^,^22^. For intracellular pathogens that target innate immune cells and are associated with severe and highly threatening chronic diseases, such as *Chlamydia trachomatis*, *Coxiella burnetii* and *Mycobacterium tuberculosis*, it is predictable that these pathogens encode unique defence mechanisms to subvert inflammasome activation. In fact, factors that inhibit inflammasome activation have been identified in *M. tuberculosis* and in *C. trachomatis*, although the molecular mechanisms of inflammasome inhibition and host cell targets are unclear^23^,^24^,^25^.

The intracellular bacterial pathogen *C. burnetii* is the causative agent of an acute pneumonia-like disease called Q fever, which may lead to endocarditis or hepatitis^26^. The bacteria replicate within a phagolysosomal vacuole and infect primarily alveolar macrophages (AMs)^27^. *C. burnetii* is highly specialized to subvert host cell functions, including the avoidance of TLRs recognition, the inhibition of apoptosis and the modulation of diverse vesicle traffic pathways^28^,^29^,^30^,^31^,^32^,^33^,^34^,^35^,^36^,^37^,^38^,^39^. However, immunocompetent individuals can effectively control bacterial multiplication, thus emphasizing that effective immune responses are critical for host resistance. *C. burnetii* is evolutionarily close to *L. pneumophila*, belonging to the order of Legionellales. Importantly, these pathogens share a homologous Dot/Icm type IV secretion system^40^. Historically, the genetic manipulation of *C. burnetii* has not been easily achieved, and as such, *L. pneumophila* has been used as a surrogate host to characterize and identify the *C. burnetii* Dot/Icm secretion system^41^,^42^. *C. burnetii* encodes diverse putative effector proteins in its genome, some of them with sequence motifs predicted to mediate eukaryotic protein–protein interactions such as ankyrin repeat-containing and leucine-rich repetition-containing domains^40^,^43^. More than 100 putative *C. burnetii* effectors identified by genetic and bioinformatic whole-genome screens have been shown to be secreted by the Dot/Icm system of *L. pneumophila*^44^,^45^,^46^,^47^,^48^,^49^,^50^. Despite this massive identification of the *C. burnetii* effectors, the molecular function of very few effector proteins has been defined. Because *C. burnetii* is a highly virulent intracellular pathogen and very few cells (<5 organisms) are sufficient to induce disease in a healthy human being, we investigated whether inflammasome activation occurs in response to *C. burnetii* infection. We found that *C. burnetii* inhibits the non-canonical activation of the NLRP3 inflammasome, which occurs in a process dependent on caspase-11 and independent of NLRC4 and bacterial flagellin. To search for the mechanism underlying the inhibition of the inflammasome by the bacterium, we screened potential effectors using *L. pneumophila* as a surrogate host and identified a *C. burnetii* effector protein, denoted herein as IcaA (*Inhibition of caspase activation*), that interferes with the caspase-11-mediated, non-canonical activation of the inflammasome. *C. burnetii* mutants for *icaA* fail to inhibit caspase-11 activation induced by *flaA* mutants of *L. pneumophila*. These findings unveil the process of inflammasome inhibition by *C. burnetii* and therefore contribute to our understanding of the molecular mechanisms underlying the virulence strategies used by this highly threatening obligate intracellular pathogen.

## Results

### *C. burnetii* infection does not stimulate inflammasome activation

**Figure 1:**
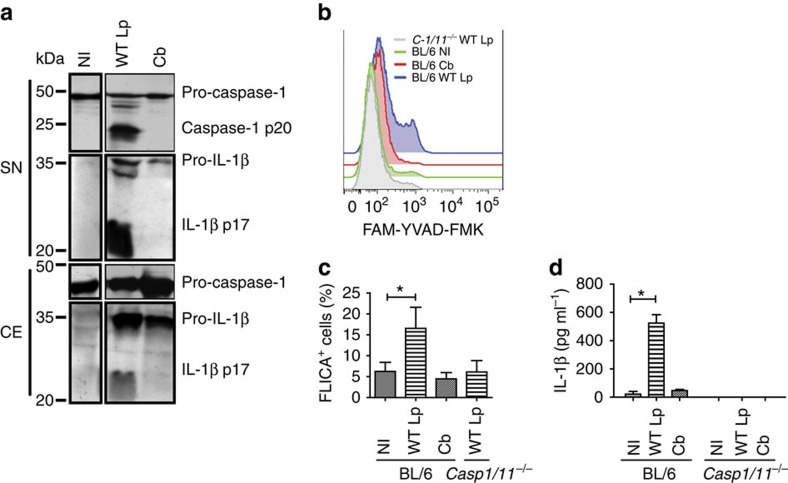
Caspase-1 is not activated in macrophages infected with *C. burnetii*. Bone marrow-derived macrophages (BMDMs) remained noninfected (NI) or were infected with wild-type *L. pneumophila* for 9 h (WT Lp, multiplicity of infection (MOI) 10) or *C. burnetii* for 24 h (Cb, MOI 30). (**a**) Immunoblot showing processed p20 subunit of caspase-1 (caspase-1 p20), unprocessed caspase-1 (pro-caspase-1), p17 subunit of mature IL-1β (IL-1β p17) and pro-IL-1β, as determined in the supernatant (SN) and in the cell extract (CE). (**b**) Flow analysis of FLICA staining in BL/6 BMDM noninfected (green curve), infected with Cb (red curve) or infected with WT Lp (blue curve) and *Casp1/11*^*−/−*^ BMDMs infected with WT Lp (grey curve). (**c**) Quantification of FLICA-positive cells in (**b**). (**d**) IL-1β secretion in infected BMDMs was determined by ELISA. Data in (**c**,**d**) are expressed as average±s.e.m. of triplicate wells and significance was calculated with *t*-test. *P<0.05 compared with NI cells. Data are representative of at least three independent experiments. Full blots are presented in the Supplementary Fig. 10.

To investigate whether *C. burnetii* induces caspase-1 activation, we infected murine bone marrow-derived macrophages (BMDMs) for 24 h and assessed the processing of caspase-1 and pro-IL-1β by western blot. We found that the infection of wild-type C57BL/6 BMDMs (BL/6 BMDMs) with *C. burnetii* does not induce the cleavage of caspase-1 p20 subunit (Fig. 1a). As a control, we used *L. pneumophila*, which readily triggers the processing of these proteins in infected BMDMs (Fig. 1a). To further evaluate caspase-1 activation in response to *C. burnetii*, we assessed caspase-1 activation in BMDMs using the FAM-YVAD-FMK (FLICA), a previously described fluorescent probe that specifically binds the active form of caspase-1 while inhibiting its autoproteolytic processing^11^. We found that in contrast to *L. pneumophila* infection, *C. burnetii* infection does not induce an increase in the percentage of cells stained with FLICA, as measured by flow cytometry (Fig. 1b,c). As a control, we used BMDMs from a *Casp1/11*^*−/−*^ mouse to indicate that this probe is specific to caspase-1. Finally, we measured the secretion of IL-1β as readout for caspase-1 activation. We found that as opposed to *L. pneumophila* infection, *C. burnetii* infection does not induce the secretion of IL-1β (Fig. 1a,d). Altogether, these data show that *C. burnetii* does not trigger caspase-1 activation in BMDMs.

### *C. burnetii* inhibits NLRP3 activation induced by caspase-11

**Figure 2:**
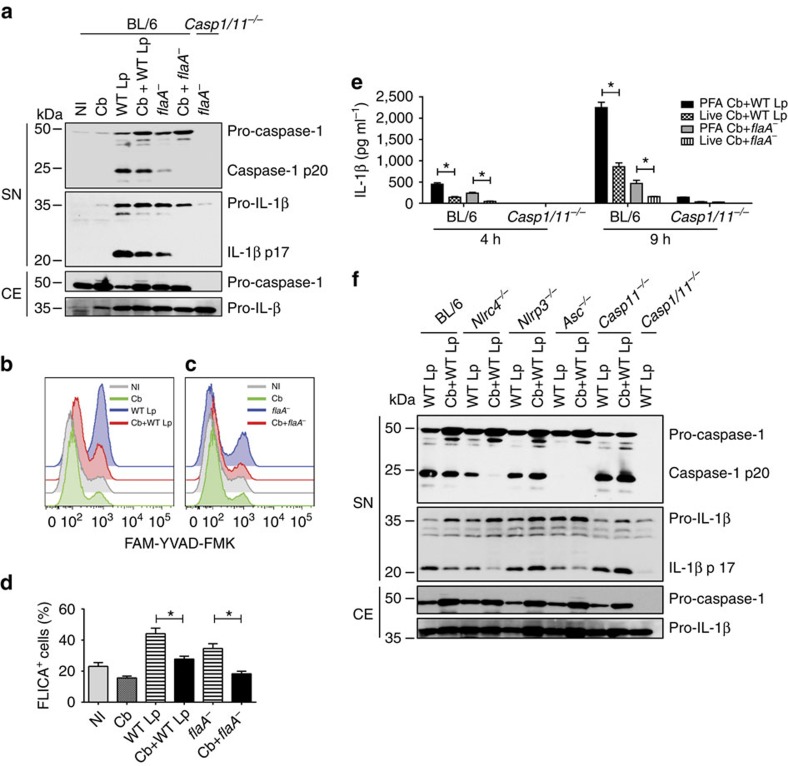
*C. burnetii* inhibits the caspase-11-mediated non-canonical activation of the NLRP3 inflammasome induced by *L. pneumophila*. Bone marrow-derived macrophages (BMDMs) were left noninfected (NI) or were infected with *C. burnetii* (Cb, MOI 30) for 24 h and further infected with wild-type *L. pneumophila* (WT Lp, MOI 10) or *flaA*^*−*^ (MOI 10) for 9 h. Co-infected BMDMs are indicated (Cb+WT Lp or Cb+*flaA*^*−*^). (**a**) Immunoblot showing levels of processed p20 subunit of caspase-1 (caspase-1 p20), unprocessed caspase-1 (pro-caspase-1), p17 subunit of mature IL-1β (IL-1β p17) and pro-IL-1β, as determined in supernatant (SN) and cell extract (CE). (**b**,**c**) Flow analysis of FLICA staining in BL/6 BMDM noninfected (grey curve), infected with Cb (green curve), infected with *L. pneumophila* (blue curve) or co-infected (red curve). (**d**) Quantification of FLICA-positive cells in (**b**,**c**). (**e**) IL-1β secretion in infected BMDMs was determined by ELISA. BMDMs infected with live *C. burnetii* (live Cb) or with PFA-fixed were used. (**f**) Caspase-1 cleavage and IL-1β secretion were evaluated by immunoblot as in (**a**). Data in (**d**,**e**) are expressed as average±s.e.m. of triplicate wells and significance was calculated with ANOVA. *P<0.05. Data are representative of three (**a**,**f**) and two (**b**–**e**) independent experiments.

Although macrophages use multiple sensors to detect Gram-negative bacteria, *C. burnetii* can evade immune responses and interfere with different cellular process to establish a replicative niche in these cells. Therefore, we investigated whether the absence of caspase-1 activation results from the active process of inhibition by *C. burnetii*. Previous work has demonstrated that *C. burnetii* and *L. pneumophila* establish distinct and functional replicative vacuoles in co-infected BMDMs^51^. We thus performed co-infection studies to evaluate whether *C. burnetii* can actively inhibit the inflammasome activation triggered by *L. pneumophila*. We found that pre-infection of BMDMs with *C. burnetii* for 24 h resulted in reduced caspase-1 activation and pro-IL-1β cleavage in response to a second infection with *L. pneumophila* (Fig. 2a). Notably, pre-infection with *C. burnetii* induced a significantly higher expression of pro-casp-1 and pro-IL-1β, than single infections with *L. pneumophila*, possibly because of the presence of *C. burnetii* PAMPs during the 24-h pre-stimulation. To guarantee that the cells were stimulated similarly during the co-infection experiments, we pre-infected BMDMs with live or paraformaldehyde (PFA)-fixed *C. burnetii* before infections with *L. pneumophila*. We found that PFA-inactivated *C. burnetii* induced a similar expression of pro-casp-1 as the live bacteria, but only live *C. burnetii* inhibited caspase-1 activation in response to *L. pneumophila* (Supplementary Fig. 1a). These data indicate that the inhibition of caspase-1 activation is an actively induced process that requires *C. burnetii* metabolic activity. Caspase-1 activation in response to *L. pneumophila* occurs via at least two distinct pathways, one dependent on and another independent of bacterial flagellin^52^. To evaluate whether *C. burnetii* inhibits the flagellin-dependent or -independent pathway triggered by *L. pneumophila* infection, we performed co-infections of *C. burnetii* with wild-type (WT Lp) or flagellin-deficient (*flaA*^*−*^) *L. pneumophila*. We found that *C. burnetii* pre-infection inhibits caspase-1 activation and IL-1β processing in response to either WT Lp or *flaA*^*−*^ *L. pneumophila* (Fig. 2a). Strikingly, the co-infections performed with *flaA*^*−*^ *L. pneumophila* revealed that the flagellin-independent pathway for caspase-1 activation was abolished when the BMDMs were pre-infected with *C. burnetii* (Fig. 2a). These data indicate that *C. burnetii* effectively inhibits the flagellin-independent pathway for caspase-1 activation. Similar experiments performed with PFA-fixed *C. burnetii* indicated that live *C. burnetii* is required for the inhibition of caspase-1 activation in response to *flaA*^*−*^ *L. pneumophila* (Supplementary Fig. 1b).

These findings were further confirmed by the assessment of endogenous caspase-1 activation with the FLICA assay. BMDMs pre-infected with *C. burnetii* showed a reduction in caspase-1 activation in response to infection with both the WT Lp and *flaA*^*−*^ *L. pneumophila* (Fig. 2b–d). As demonstrated by western blot, when co-infections were performed using *flaA*^*−*^ *L. pneumophila*, we found that BMDMs do not trigger caspase-1 activation (Fig. 2c,d). Pre-infections with *C. burnetii* did not reduce the internalization of *L. pneumophila* in BMDMs (Supplementary Fig. 2), confirming that this phenomenon is not caused by differences in *L. pneumophila* internalization after a previous exposure to *C. burnetii*. The *C. burnetii*-mediated inhibition of caspase-1 activation induced by *L. pneumophila* was also assessed by measuring the secretion of active IL-1β by enzyme-linked immunosorbent assay (ELISA). We found that pre-infection with live *C. burnetii* inhibited the secretion of IL-1β in response to infection with WT Lp, and abolished the secretion of IL-1β in response to infection with *flaA*^*−*^ *L. pneumophila* (Fig. 2e).

**Figure 3:**
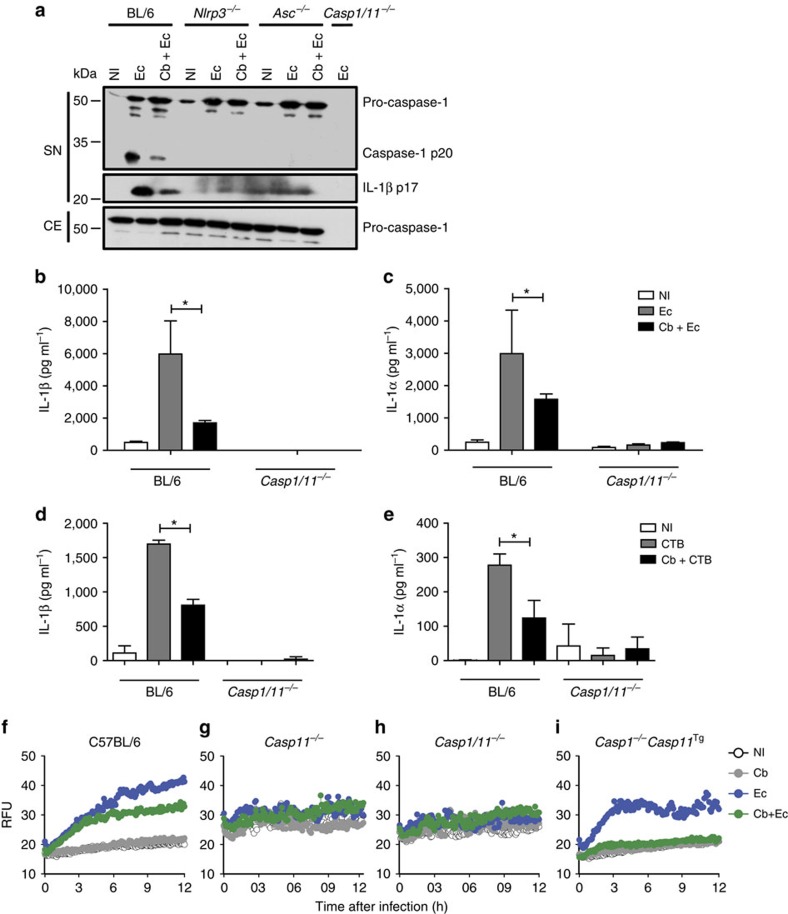
*C. burnetii* inhibits caspase-11-dependent pore formation and NLRP3 activation in response to *E. coli* and cholera toxin B. Bone marrow-derived macrophages (BMDMs) generated from wild-type C57BL/6 (BL/6), *Nlrp3*^*−/−*^, *Asc*^*−/−*^ or *Casp1/11*^*−/−*^ mice were left noninfected (NI) or were infected with *C. burnetii* (Cb, MOI 30) for 24 h, stimulated with LPS (0.5 μg ml^−1^) for 4 h and infected with *E. coli* at MOI 20 (Ec or Cb+Ec) or treated with CTB at 20 μg ml^−1^ (CTB or Cb+CTB). (**a**) Immunoblot showing levels of processed p20 subunit of caspase-1 (caspase-1 p20), unprocessed caspase-1 (pro-caspase-1) and p17 subunit of mature IL-1β (IL-1β p17), as determined in supernatant (SN) and cell extract (CE) after 12 h of infection with *E. coli.* (**b**) IL-1β and (**c**) IL-1α were determined by ELISA from cell supernatants 16 h after *E. coli* infection. (**d**) IL-1β and (**e**) IL-1α were determined by ELISA from cell supernatants 16 h after CTB stimulation. (**f**–**i**) Pore formation was assessed fluorometrically in real time by the uptake of propidium iodide (RFUs, relative fluorescence units). (**b**–**e**) Data are expressed as the average±s.e.m. of triplicate wells and significance was calculated with ANOVA. *P<0.05. (**a**–**i**) Data are representative of two independent experiments.

We investigated whether *C. burnetii* is able to inhibit caspase-1 activation in response to other stimuli. Thus, BMDMs pre-infected or not with *C. burnetii* were stimulated with LPS+ATP or with LPS+dsDNA to trigger the activation of the inflammasomes dependent on NLRP3 and AIM2, respectively. We found that *C. burnetii* fails to inhibit caspase-1 activation in response to ATP (Supplementary Fig. 3a,c) and dsDNA (Supplementary Fig. 3b,d). These results argue that *C. burnetii* does not directly inhibit caspase-1 activation but may interfere with upstream events in the signalling cascade of the inflammasome triggered by *L. pneumophila*. It was recently demonstrated that *L. pneumophila* triggers the non-canonical activation of the NLRP3 inflammasome in a process independent of flagellin and dependent on caspase-11 (ref. 13). Thus, we used BMDMs from *Nlrc4*^*−/−*^, *Nlrp3*^*−/−*^, *Asc*^*−/−*^ or *Casp11*^*−/−*^ mice to evaluate the requirement of these proteins for the *C. burnetii*-mediated inhibition of caspase-1 activation in response to *L. pneumophila*. We found that the inhibition of caspase-1 processing occurs in *Nlrc4*^*−/−*^ BMDMs, a feature that is consistent with the findings that *C. burnetii* inhibits the flagellin-independent pathway for caspase-1 activation (Fig. 2f). In the absence of ASC, we detected no caspase-1 processing. In *Nlrp3*^*−/−*^ and *Casp11*^*−/−*^ BMDMs, we found that *C. burnetii* fails to inhibit caspase-1 cleavage and the processing of pro-IL-1β (Fig. 2f). This feature is consistent with the hypothesis that *C. burnetii* inhibits the non-canonical activation of the NLRP3 inflammasome, which occurs through caspase-11 and culminates in the activation of the NLRP3/ASC/caspase-1 platform, leading to caspase-1 cleavage and IL-1β production^53^. It was previously demonstrated that Gram-negative bacteria, such as *E. coli*, or the combination of LPS plus the B subunit of cholera toxin (CTB) triggers the non-canonical activation of the inflammasome^18^,^53^. Thus, to test whether *C. burnetii* inhibits the non-canonical activation of the inflammasome, we pre-infected BMDMs with *C. burnetii*, stimulated with either LPS+CTB or *E. coli* and measured caspase-1 activation. We found that *C. burnetii* pre-infection severely inhibited caspase-1 activation and pro-IL-1β processing in response to *E. coli* (Fig. 3a). According to previous reports, the *E. coli*-induced activation of caspase-1 and pro-IL-1β cleavage was dependent on NLRP3 and ASC (Fig. 3a). Supporting the inhibitory activity of *C. burnetii* in the non-canonical inflammasome activation, we found that the release of IL-1β and IL-1α in response to *E. coli* or LPS+CTB is reduced in BMDMs pre-infected with *C. burnetii* (Fig. 3b–e). Next, we used a pore formation assay that reflects a caspase-11 activity that operates independently of NLRC4 inflammasome and upstream of the non-canonical activation of the NLRP3 inflammasome^13^,^54^. By co-infecting cells with *C. burnetii* and *E. coli*, we found that *C. burnetii* is able to inhibit the caspase-11-mediated pore formation induced by *E. coli* (Fig. 3f–h). In this experiment, we also included BMDMs from a mouse deficient in caspase-1. This mouse, herein called *Casp1*^*−/−*^*/Casp11*^*Tg*^, is the *Casp1/11*^*−/−*^ expressing a transgene encoding a functional copy of the caspase-11 allele, as described previously^53^. By using *Casp1*^*−/−*^*/Casp11*^*Tg*^ BMDMs, we confirmed that *C. burnetii* is able to inhibit caspase-11-mediated pore formation induced by *E. coli* in the absence of caspase-1 (Fig. 3i). Next, we tested whether the inhibition of caspase-11 also operates in primary mouse AMs, which are permissive for *C. burnetii* replication, and are more similar to the relevant cells that harbour *C. burnetii* during acute Q fever (Supplementary Fig. 4a). By using AMs, we found that co-infections with live *C. burnetii* but not with heat-killed bacteria inhibit the caspase-11-dependent pore formation induced by *E. coli* in primary AMs similarly to BMDMs (Supplementary Fig. 4b,c).

### A genetic screen using *L. pneumophila* identifies *C. burnetii* IcaA

We used *L. pneumophila* as a surrogate host to screen the specific *C. burnetii* effector proteins involved in the inhibition of non-canonical inflammasome activation, and we constructed a library of *flaA*^*−*^ mutants of *L. pneumophila* expressing *C. burnetii* effectors that have been confirmed to be secreted via the *L. pneumophila* type IV secretion system^44^,^45^. We obtained *L. pneumophila* clones with a confirmed expression of 24 *C. burnetii* effectors (Supplementary Fig. 5a,b). *L. pneumophila* mutants expressing *C. burnetii* genes were screened by western blot for the induction of caspase-1 cleavage in infected BMDMs. This screening identified a strain that induced neither caspase-1 activation nor IL-1β maturation, which was the clone of *L. pneumophila* that produces the effector protein annotated as CBU1823 (Supplementary Fig. 5c–e). Although biochemical activities displayed by the CBU1823 protein are yet unknown, this protein has been shown to be secreted into host cells through the *L. pneumophila* Dot/Icm system^44^,^45^. In addition, a recent report demonstrated that CBU1823 is translocated into BMDMs during *C. burnetii* infection in a process dependent on the *C. burnetii* Dot/Icm system^55^. Because we identified CBU1823 as a *C. burnetii* Dot/Icm effector protein that inhibits caspase activation, we named this protein IcaA. IcaA shows no known protein domain and has no significant homology to any other eukaryotic or bacterial protein to hint at its mechanisms of function.

We further tested whether the expression of IcaA in different strains of *flaA*^*−*^ *L. pneumophila* displayed reduced caspase-1 cleavage in BMDMs. We found that the inhibition of caspase-1 cleavage by IcaA occurred in both JR32 and Lp01 backgrounds (Supplementary Fig. 6a). Inhibition also occurred when we used the strain Lp02, which is a thymidine auxotroph and does not replicate intracellularly in the absence of thymidine supplementation (Supplementary Fig. 6a). Hence, IcaA-mediated caspase-1 inhibition is independent of bacterial replication. Next, we investigated whether the expression of IcaA influenced *L. pneumophila* fitness. We compared *flaA*^*−*^ mutants of *L. pneumophila* expressing IcaA (*flaA*^*−*^/pIcaA) with those encoding the empty vector (*flaA*^*−*^/pVec) in assays that measured replication in axenic media, internalization in BMDMs and the induction of cytokines. We found that the expression of IcaA does not affect the bacterial replication in liquid ACES-buffered yeast extract (AYE) axenic media, bacterial internalization in BMDMs or the induction of IL-12 in BMDMs (Supplementary Fig. 6b–d). Moreover, the expression of IcaA or the unrelated *C. burnetii* effector protein AnkH in *flaA*^*−*^ *L. pneumophila* did not interfere with internalization or the early bacterial replication in BMDMs at 9 h of infection compared with the bacteria encoding the empty vector (Supplementary Fig. 6e,f). The bacteria encoding pIcaA, but not those encoding the pVec, showed a reduced activation of caspase-1 and secretion of IL-1β (Supplementary Fig. 6a,g).

**Figure 4:**
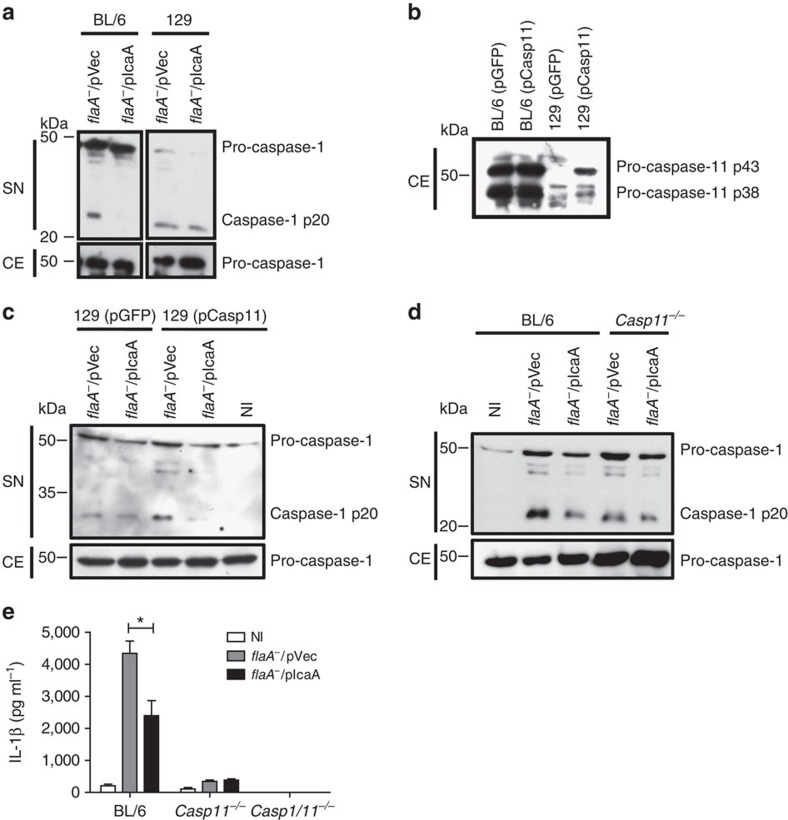
IcaA requires a functional caspase-11 to inhibit caspase-1 activation. Bone marrow-derived macrophages (BMDMs) generated from C57BL/6 (BL/6) and 129S6/SvEv (129) mice were left noninfected (NI) or were infected with *flaA*^*−*^ mutants of *L. pneumophila* that were transformed either with an empty vector (*flaA*^*−*^/pVec) or the vector encoding IcaA (*flaA*^*−*^*/*pIcaA) at MOI 10 for 9 h. (**a**) Immunoblot showing processed p20 subunit of caspase-1 (caspase-1 p20) and unprocessed caspase-1 (pro-caspase-1), as determined in supernatant (SN) and cell extract (CE). (**b**) Immunoblot showing the expression of unprocessed isoforms of pro-caspase-11 (p43 and p38) in uninfected BL/6 and caspase-11-deficient BMDMs derived from 129 mouse strain were transduced with the pMSCV virus encoding GFP (pGFP) or pMSCV encoding full-length caspase-11 (pCasp-11). The expression of unprocessed isoforms of pro-caspase-11 (p43 and p38) is shown by western blot. (**c**) Immunoblot showing caspase-1 p20 and pro-caspase-1 as determined in SN and CE from transduced BMDMs infected for 9 h. (**d**) Immunoblot showing caspase-1 p20 and pro-caspase-1 in SN and CE of BMDMs primed with LPS (0.5 μg ml^−1^) and infected for 3 h. (**e**) IL-1β was determined by ELISA from SN of BMDMs primed with LPS for 3 h. Data are expressed as the average±s.e.m. of triplicate wells and significance was calculated with ANOVA. *P<0.05. Data are representative of at least two (**b**,**c**) or three (**a**,**d**,**e**) independent experiments.

Our studies, using pre-infections with live *C. burnetii*, supported the findings that *C. burnetii* inhibits the non-canonical activation of the inflammasome. Therefore, we used the *flaA*^*−*^ *L. pneumophila*-carrying pIcaA (or pVec) to evaluate whether IcaA is involved in the inhibition of the caspase-11-mediated, non-canonical activation of the NLRP3 inflammasome. Initially, we investigated whether caspase-11 is required for the IcaA-mediated inhibition of caspase-1 activation. We found that whereas *flaA*^*−*^ mutants encoding pVec trigger caspase-1 activation in BL/6 BMDMs, the mutants expressing IcaA fail to do so. Importantly, the IcaA-mediated inhibition of caspase-1 activation is not observed in BMDMs from mice of a 129S6/SvEv genetic background (herein called 129), a strain that fails to express functional caspase-11 (Fig. 4a,b). To certify that the lack of caspase-1 inhibition in 129 mouse strains was due to the absence of caspase-11, we transduced 129 BMDMs with a retrovirus-encoding caspase-11 (or GFP as a control). The transduced cells expressed a significant amount of p43 and p38 isoforms of pro-caspase-11 (Fig. 4b). By infecting the transduced cells, we found that *flaA*^*−*^ *L. pneumophila* expressing pIcaA inhibits the caspase-1 cleavage in 129 BMDMs expressing caspase-11 but not in cells transduced with retrovirus-encoding GFP (Fig. 4c). In agreement with these findings, we found that *flaA*^*−*^/pIcaA mutants do not inhibit caspase-1 cleavage or IL-1β secretion in LPS-primed *Casp11*^*−/−*^ BMDMs in the BL/6 genetic background (Fig. 4d,e). In contrast, in the BL/6 BMDMs, IcaA expression partially inhibited caspase-1 activation and IL-1β secretion induced by *flaA*^*−*^ *L. pneumophila*, which are convenient proxy measures for caspase-11 activation (Fig. 4d,e). Supporting the role of caspase-11 in the flagellin-independent pathway for caspase-1 activation, we found that BMDMs from *Casp11*^*−/−*^ mice show a reduced activation of caspase-1 and secretion of IL-1β compared with BL/6 BMDMs (Fig. 4d,e).

### IcaA inhibits caspase-11-mediated inflammasome activation

**Figure 5:**
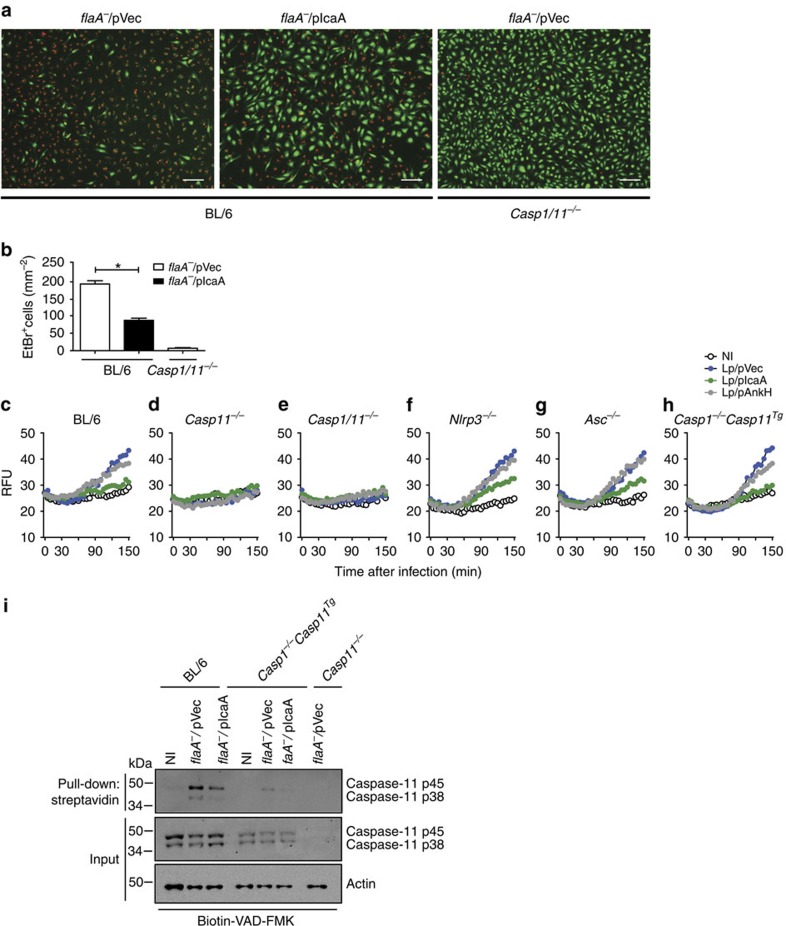
IcaA does not inhibit caspase-11 activation directly but inhibits a caspase-11 activation upstream of the non-canonical activation of the NLRP3 inflammasome. (**a**–**i**) Bone marrow-derived macrophages (BMDMs) were primed with LPS (0.5 μg ml^−1^) for 3 h, remained noninfected (NI) or were infected with *flaA*^*−*^ *L. pneumophila* transformed either with an empty vector (*flaA*^*−*^/pVec), the vector encoding IcaA (*flaA*^*−*^/pIcaA), or the vector encoding AnkH (*flaA*^*−*^/pAnkH) at MOI 10. (**a**) Fluorescence micrographs revealing cell permeability, as assessed by quantification of ethidium bromide influx (red). Healthy cells are stained with acridine orange (green). Scale bar, 75 μm. (**b**) Quantification of the experiment shown in (**a**). Data are expressed as the average±s.e.m. of triplicate wells and significance was calculated with *t*-test. *P<0.05. (**c**–**h**) Pore formation was assessed fluorometrically in real time by the uptake of propidium iodide (RFUs, relative fluorescence units). (**i**) Immunoblot showing the presence of caspase-11 p45 and p38 in the total cell lysate (input) and pull-down fraction using the agarose-streptavidin fraction of BMDMs treated with biotin-VAD-FMK 15 min before infection. Actin (β-actin) was used as a loading control. Data are representative of three (**a**–**h**) and two (**i**) independent experiments.

Our data reported thus far indicate that IcaA is involved in the inhibition of a caspase-11-dependent, non-canonical activation of inflammasomes. Therefore, we tested whether IcaA interferes with a caspase-11-dependent process that is upstream of caspase-1. We have previously reported that caspase-11 is involved in the formation of pores in the macrophage membrane that are independent of NLRP3, ASC and caspase-1 (ref. 13). Therefore, we investigated whether *flaA*^*−*^ mutants expressing IcaA interfere with caspase-11-mediated pore formation in BMDMs. By analysing the uptake of cell impermeable dye ethidium bromide, we found a partial reduction in pore formation in BMDMs infected with *flaA*^*−*^/pIcaA bacteria in comparison to those infected with *flaA*^*−*^/pVec (Fig. 5a,b). Next, we performed analyses using the less toxic dye propidium iodide (PI) to evaluate the kinetics of pore formation in real time. By using this approach, we confirmed that IcaA, but not the unrelated *C. burnetii* effector AnkH, partially inhibits the caspase-11-dependent pore formation observed in response to *flaA*^*−*^ *L. pneumophila* (Fig. 5c). Pore formation was not detected in *Casp11*^*−/−*^ or *Casp1/11*^*−/−*^ BMDMs, emphasizing that the flagellin-independent pore formation is caspase-11-dependent (Fig. 5d,e). Notably, the IcaA-mediated inhibition of pore formation occurred normally in BMDMs from *Nlrp3*^*−/−*^, *Asc*^*−/−*^ and *Casp1*^*−/−*^*/Casp11*^*Tg*^ mice (Fig. 5f–h). These data further confirm that IcaA interferes with caspase-11-mediated responses but not in the downstream components of the NLRP3 inflammasome, such as NLRP3, ASC and caspase-1. To further evaluate whether the IcaA-mediated inhibition of pore formation is specific to caspase-11, we expressed IcaA in flagellated *L. pneumophila*, which is known to trigger pore formation by a process that is Nlrc4- and caspase-1-dependent but caspase-11-independent^10^,^13^,^52^,^54^,^56^,^57^. By infecting BMDMs from BL/6, *Casp11*^*−/−*^ and *Casp1/11*^*−/−*^ mice with *flaA*^*+*^/pIcaA or *flaA*^*+*^/pVec, we found that the IcaA is not effective in inhibiting the flagellin-mediated pore formation that is dependent on caspase-1 but independent of caspase-11 (Supplementary Fig. 7a). As expected, infections performed with *flaA*^*−*^/pIcaA inhibited caspase-11-dependent pore formation compared with *flaA*^*−*^/pVec (Supplementary Fig. 7b). All these data indicate that IcaA is specifically involved in the inhibition of caspase-11-mediated processes, but it does not influence caspase-1-mediated processes that do not require caspase-11. Our assertion that *C. burnetii* inhibits a caspase-11-dependent pathway implies that the bacteria express molecules that trigger this pathway. To evaluate whether *C. burnetii* is able to trigger caspase-11 activation, we extracted *C. burnetii* LPS and tested for the induction of pore formation upon the transfection of BMDMs. We detected a robust pore formation in BMDMs transfected with *C. burnetii* LPS, which was reduced in *Casp1*^*−/−*^/*Casp11*^*Tg*^ and *Casp11*^*−/−*^ BMDMs (Supplementary Fig. 8). These data support the hypothesis that *C. burnetii* expresses molecules that trigger pore formation if delivered in the macrophage cytoplasm, a feature that supports the development of effectors, such as IcaA, to inhibit this response.

Our data generated thus far indicate that IcaA is an effector protein encoded by *C. burnetii* that mediates the inhibition of caspase-11 functions such as pore formation, but it is not clear whether IcaA inhibits caspase-11 activation. Thus, we tested whether IcaA-expressing *flaA*^*−*^ *L. pneumophila* can reduce the activation of caspase-11 in the infected BMDMs. To measure caspase-11 activation directly, we pulled down active caspase-11 from macrophages lysates using biotin-VAD-FMK. Active caspase-11-bound to biotin-VAD was concentrated with streptavidin, and the samples were blotted with anti-caspase-11. We detected a robust caspase-11 activation when BL/6 BMDMs were infected with *flaA*^*−*^ *L. pneumophila* (Fig. 5i). Notably, IcaA expression in *L. pneumophila* reduced caspase-11 activation compared with the bacteria expressing the empty vector (Fig. 5i). The IcaA-mediated inhibition of caspase-11 activation was also observed in experiments performed with macrophages from the *Casp1*^*−/−*^*/Casp11*^*Tg*^ mice (Fig. 5i). Notably, the overall expression of caspase-11 in these cells is lower than that of C57BL/6 macrophages, possibly because only one copy of the *Casp11*^*tg*^ is present in the genome. Regardless of the reduced expression of caspase-11 in the *Casp1*^*−/−*^*/Casp11*^*Tg*^ BMDMs, these data further confirm our assertion that IcaA is involved in the inhibition of caspase-11 activation despite the presence of caspase-1 (Fig. 5e).

### *icaA* ^*-*^ *C. burnetii* fails to inhibit inflammasome activation

**Figure 6:**
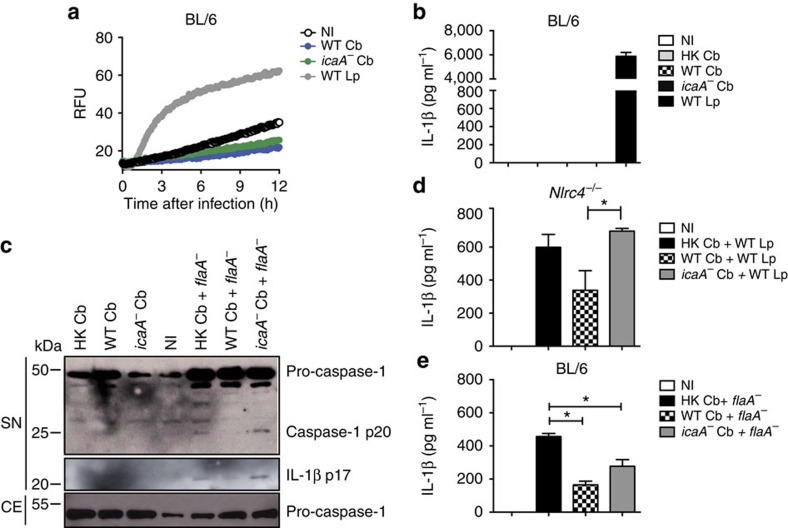
*C. burnetii* mutants for *icaA* fail to inhibit the non-canonical activation of the inflammasome in response to *flaA*^*−*^ *L. pneumophila*. Bone marrow-derived macrophages (BMDMs) were left noninfected (NI) or were infected with heat-killed wild-type (HK Cb) wild-type (WT Cb) or *icaA*^*-*^ mutant (*icaA*^*-*^ Cb) of *C. burnetii* for 24 h at MOI 30, and further infected with wild-type *L. pneumophila* (WT Lp, MOI 10) or *flaA*^*−*^ (MOI 10) for 9 h. Co-infected BMDMs are indicated (Cb+Lp or Cb+*flaA*^*−*^). (**a**) Pore formation was assessed fluorometrically in real time by the uptake of propidium iodide (RFUs, relative fluorescence units). (**b**,**d**,**e**) IL-1β secretion in infected BMDMs was determined by ELISA. Data are expressed as the average±s.e.m. of triplicate wells and significance was calculated with *t*-test. *P<0.05. (**c**) Immunoblot showing processed p20 subunit of caspase-1 (caspase-1 p20), unprocessed caspase-1 (pro-caspase-1) and p17 subunit of mature IL-1β (IL-1β p17), as determined in supernatant (SN) and cell extract (CE). (**a**–**e**) Data are representative of at least two independent experiments.

To further evaluate whether the endogenously expressed IcaA in *C. burnetii* is involved in the inhibition of the inflammasome activation, we generated the *icaA*^*-*^ mutant *C. burnetii* (Supplementary Fig. 9). Initially, we tested whether *icaA*^*-*^ is able to trigger caspase-11-dependent responses in single macrophage infections. Measuring caspase-11-dependent pore formation in cells infected with *C. burnetii*, we found that neither the wild-type *C. burnetii* nor the *icaA*^*-*^ mutants triggered a caspase-11-dependent pore formation (Fig. 6a). Accordingly, *icaA*^*-*^ mutants failed to trigger the caspase-11-mediated non-canonical activation of the NLRP3 inflammasome, as measured by the production of IL-1β (Fig. 6b). These data suggest that additional *C. burnetii* processes may be involved in the activities related to the inhibition of inflammasome activation by this bacterium. Next, we tested whether IcaA expression is required for the inhibition of non-canonical inflammasome activation in co-infection experiments. In these experiments, *Nlrc4*^*−/−*^ BMDMs or *flaA*^*−*^ *L. pneumophila* were used to bypass the activation of NLRC4 inflammasome. We found that, whereas a single infection with wild-type *C. burnetii* or with the *icaA*^*-*^ mutants did not trigger caspase-1 activation in BMDMs, the co-infection with *icaA*^*-*^ *C. burnetii* failed to suppress the caspase-1 activation induced by *flaA*^*−*^ *L. pneumophila* (Fig. 6c). Accordingly, co-infections performed with wild-type *C. burnetii*, but not with *icaA* mutants, inhibited the caspase-11-mediated non-canonical activation of the inflammasome, as measured by IL-1β production (Fig. 6d,e). These data further confirm that endogenously expressed IcaA in *C. burnetii* is important for the activities related to the inhibition of the caspase-11-mediated non-canonical activation of the inflammasome.

## Discussion

**Figure 7:**
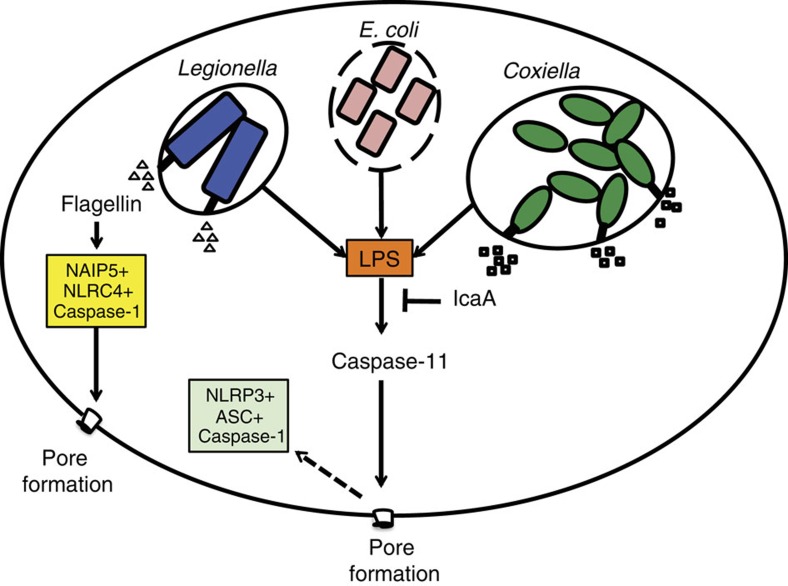
Model for IcaA-mediated inhibition of non-canonical activation of the inflammasome. Published data demonstrate that caspase-11 senses the presence of cytosolic LPS from Gram-negative bacteria, including *E. coli* and *L. pneumophila*. Activation of caspase-11 in response to *L. pneumophila* requires the expression of Dot/Icm. The effector protein IcaA is translocated by the *C. burnetii* Dot/Icm and inhibits the caspase-11-mediated pore formation and caspase-11-induced non-canonical activation of the NLRP3 inflammasome.

It is estimated that as few as five infectious *C. burnetii* organisms are able to establish infection in healthy individuals exposed to the bacteria^58^. To achieve such a high infectious efficiency, *C. burnetii* inhibits a large repertoire of specific host cell responses, including those related to the innate immune activation and induction of host cell death. Although the inhibition of caspase-3-mediated apoptosis by *C. burnetii* has been reported previously^29^,^31^,^32^,^35^, an evaluation of inflammatory caspases and pyroptosis has never been conducted. Herein, we report that *C. burnetii* fails to induce caspase-1 activation upon the infection of primary mouse macrophages. We found that the bacteria actively inhibit the caspase-11 activation and their functions including pore formation and the non-canonical activation of the NLRP3 inflammasome, but they do not inhibit the components of inflammasomes such as ASC, NLRP3 or caspase-1. By searching for the molecular mechanisms underlying this process, we identified a novel *C. burnetii* gene that encodes an effector protein translocated by the Dot/Icm type IVB secretion system into host cell cytosol. We named this protein IcaA because it is functionally related to the inhibition of caspase activation. IcaA suppresses the caspase-11-mediated, non-canonical activation of caspase-1 in macrophages. Notably, we have no data to support a direct effect of IcaA in the inhibition of caspase-11 activation. Most likely, IcaA is involved in the inhibition of certain cellular processes, which leads to caspase-11 activation (Fig. 7). It is also possible that IcaA operates in the vacuolar membranes by minimizing the release of bacterial molecules from the vacuole to the cytoplasm, thereby reducing the release of PAMPs that trigger cytosolic innate immune sensors. This hypothesis is consistent our observed ability to transfer the *C. burnetii* inhibitory activity to *L. pneumophila in trans* when IcaA was expressed in *L. pneumophila*. This was achieved because of the high homology of *C. burnetii* and *L. pneumophila* Dot/Icm^40^. In fact, *L. pneumophila* has been extensively used as an effective surrogate host to investigate *C. burnetii* pathogenesis. Earlier studies demonstrated that the structural core components of the *C. burnetii* Dot/Icm can complement *L. pneumophila* mutants deficient in the respective homologous gene^41^,^42^. Further studies benefited from the high homology between the two Dot/Icm and used *L. pneumophila* to actively translocate *C. burnetii* effector proteins^44^,^45^,^46^,^47^,^48^,^49^,^50^. This approach supported the identification of several effector proteins encoded by *C. burnetii*, including the protein encoded by gene CBU1823 (refs 44, 45). However, only recently has the secretion of *C. burnetii* effectors via *C. burnetii* Dot/Icm been efficiently detected experimentally^45^. Notably, CBU1823 (IcaA) was one protein that has been used to demonstrate the functional activities of the *C. burnetii* Dot/Icm; therefore, IcaA is a bona fide effector secreted by the *C. burnetii* Dot/Icm system into the host cell cytoplasm^55^. These data demonstrate that IcaA is a bona fide substrate of the *C. burnetii* Dot/Icm, which can be translocated by the *Legionella* Dot/Icm or by the *Coxiella* Dot/Icm^44^,^45^,^55^.

The genetic manipulation of *C. burnetii* has only recently been achieved^44^,^59^. Therefore, little information is available regarding the role of specific *C. burnetii* effector proteins. It is believed that the bacterial effectors are determinants in the pathogenesis of this bacterium. A functional Dot/Icm system is required for *C. burnetii* growth in macrophages and in permissive cells, supporting an essential role of translocated effector proteins for bacterial survival^44^,^59^. By random transposon mutagenesis, several putative effectors have been shown to be important for bacterial replication^48^. Moreover, the deletion of CvpA, an effector that interacts with components of clathrin-coated endocytic vesicles, impairs the maturation of the parasitophorous vacuole and bacterial growth^30^. The gene-encoding IcaA studied herein was identified in the Nine Mile strain of *C. burnetii*, which is representative of acute disease isolates. Analysis of the genome of other important *C. burnetii* strains showed that this effector is conserved in the Dugway^44^, a strain isolated from rodents that is weakly pathogenic for guinea pigs. Notably, IcaA is encoded in truncated forms in the human endocarditis isolates K (Q154) and G (Q212) strains^44^. Whether the IcaA is non-functional in K and G strains due to frameshift mutations and whether this reflects differences in the virulence and spreading of the bacteria to other tissues is a subject for future investigation. Notably, caspase-11-induced susceptibility to infection has been demonstrated in murine models of bacterial dissemination using *Salmonella typhimurium*^60^. In this context, it remains to be determined whether the IcaA-mediated inhibition of caspase-11 and pyroptosis regulates bacterial dissemination and accounts for the development of chronic versus acute Q fever. Another matter for further investigation is the effect of *icaA* in inflammasome activation within human macrophages. It was recently demonstrated that Nine Mile phase II can trigger the production of active IL-1β in primary human AMs^61^. Although we have demonstrated that the IcaA-mediated inhibition of caspase-11 activation also occurs in primary mouse AMs, a direct comparison with primary human AMs will be important for the comprehensive assessment of the role of IcaA in the bacterial pathogenesis and host responses during Q fever. Nonetheless, the fact that primary human macrophages do trigger IL-1β production^61^ supports our findings that *C. burnetii* does encode molecules responsible for the non-canonical activation of the inflammasome.

Regardless of the determination of the role of IcaA and caspase-11 in the outcome of *C. burnetii* infection *in vivo*, our study identifies IcaA as a novel bacterial substrate that participates in the inhibition of non-canonical inflammasome activation. To the best of our knowledge, this is the first bacterial effector that interferes with pyroptosis and the activation of the non-canonical inflammasome activation in macrophages^19^. By using epithelial cells, OspC3 was identified as a *Shigella flexneri* effector protein that functions as a direct inhibitor of human caspase-4 (ref. 62). This protein was shown to be important for bacterial pathogenesis using a guinea pig model of *S. flexneri* infection. However, it remains unclear whether OspC3 operates in macrophages and whether it interferes with the non-canonical activation of the inflammasome. Regardless of the effect of OspC3 in human epithelial cells, we propose that the *C. burnetii* effector protein IcaA interferes with the process involved in caspase-11 activation, consequently inhibiting non-canonical inflammasome activation in macrophages, a molecular platform that has recently emerged as major player in innate immune responses to Gram-negative bacteria^12^,^13^,^14^,^15^,^16^,^17^,^18^,^53^. *C. burnetii* does not inhibit caspase-1 or other inflammasomes such as the NAIP5/NLRC4 or the canonical NLRP3. Because *C. burnetii* does not encode genes for flagellin expression, the bacteria may not have encountered selective pressure to develop molecules that target the activation of the NAIP5/NLRC4 inflammasome. In contrast, *C. burnetii* virulence is associated with the presence of the O-antigen in its LPS containing a tetra-acylated lipid A with long fatty acid chains^39^. Because it has been recently reported that the non-canonical activation of the inflammasome proceeds through the intracellular recognition of cytosolic LPS^16^,^17^, it is possible that the *C. burnetii* LPS triggers this pathway, and the bacterium has evolutionary evolved effectors, such as IcaA, that facilitate the subversion of this important innate immune pathway. In support of this hypothesis, we found that the delivery of *C. burnetii* LPS into the macrophage cytoplasm triggers this pathway. Notably, tetra-acylated lipid A was reported to be inefficient for activating caspase-11 (ref. 16). It is possible that variations in the *Coxiella* lipid A occur according to the bacterial growth phase. Alternatively, it is possible that another bacterial molecule is responsible for triggering caspase-11-dependent pore formation. Nonetheless, the induction of pores in macrophages transfected with *C. burnetii* LPS indicates that *C. burnetii* can trigger a pathway that is inhibited by IcaA. Collectively, the identification of IcaA and the unravelling of its mechanistic functions effectively contribute to our understanding of the biology and pathogenesis of this highly infectious intracellular pathogen and provides novel molecular structures that can be used in the development of therapies for sepsis and chronic inflammatory diseases.

## Methods

### Bacterial strains

*C. burnetii* (strain Nine Mile RSA 493 phase II) were collected and purified from the infection of irradiated Vero cell monolayers in DMEM (Sigma-Aldrich) supplemented with 5% fetal bovine serum (FBS) at 37 °C in 5% CO_2_ (ref. 63). *Legionella pneumophila*. Wild-type (WT Lp) and isogenic flagellin-deficient mutants (*flaA*^*−*^) of *L. pneumophila* Lp02 (thymidine auxotrophic derivative from serogroup 1 strain Lp01)^64^ were used for co-infection assays. For the surrogate expression of *C. burnetii* effectors, WT Lp, *flaA*^*−*^ and *dotA*^*−*^ isogenic mutants from Lp01 (ref. 64) and *flaA*^*−*^ isogenic mutants of Lp02 (ref. 64) and JR32 (ref. 65) strains were transformed as described below. *L. pneumophila* was cultivated at 37 °C on MOPS-buffered charcoal-yeast extract (CYE) agar plates (1% (w/v) yeast extract, 1% (w/v) MOPS, 3.3 mM L-cysteine, 0.33 mM Fe(NO_3_)_3_, 1.5% (w/v) Bacto agar and 0.2% (w/v) activated charcoal, pH 6.9) supplemented with 100 μg ml^−1^ thymidine, as appropriate. *E. coli* (DH5α strain) was streaked at 37 °C on Luria-Bertani (LB) agar plates without antibiotics for colony isolation and cultivated in LB broth at 37 °C under rotation (200 r.p.m.).

### Mice

C57BL/6, 129S6/SvEv and C57BL/6-derived *Asc*^*−/−*^, *Casp11*^*−/−*^, *Casp1/11*^*−/−*^, *Casp1*^*−/−*^*/Casp11*^*Tg*^, *Nlrc4*^*−/−*^ and *Nlrp3*^*−/−*^ mice were bred and maintained under specific pathogen-free conditions in the Animal Facilities of the Medical School Ribeirão Preto (FMRP-USP). *Casp1*^*−/−*^*/Casp11*^*Tg*^ mice are *Casp1/11*^*−/−*^ mice expressing a transgene encoding a functional copy of the caspase-11 allele, as described previously^53^. Male or female mice with 8–12 weeks old were used. All mouse experiments were conducted according to the guidelines of the institutional committee for animal care at the Comissão de Ética em Experimentação Animal da Faculdade de Medicina de Ribeirão Preto, FMRP-USP.

### Plasmid construction and expression of Dot/Icm effectors of *C. burnetii* in *L. pneumophila*

The pSN85/Cm^+^ plasmid with a N-terminal Flag epitope tag was used to clone Dot/Icm effectors from *C. burnetii* (strain Nine Mile RSA 493 phase II). Full-length genes of effectors annotated as CBU were subcloned from pEC33 constructs^44^ through *Bam*HI and *Pst*I sites. Full-length genes of effectors annotated as Ank were amplified from purified genomic DNA^44^ by PCR with sequence-specific primers containing *Bam*HI and *Sph*I sites or *Xba*I and *Sph*I in the case of AnkB (all primers used in this study are provided in Supplementary Table 1). For AnkJ, genomic DNA from the Dugway isolate was amplified by PCR with sequence-specific primers containing *Bam*HI and *Sph*I sites. Following heat-shock transformation and amplification in *E. coli* (DH5α strain), the constructs were purified with a NucleoSpin plasmid kit (Macherey-Nagel) and subsequently used to transform *L. pneumophila* by electroporation. Transformed *L. pneumophila* clones were selected and expanded in CYE plates with 10 μg ml^−1^ chloramphenicol. Effector expression was induced with the addition of 1 mM isopropyl β-D-1-thiogalactopyranoside (IPTG, Invitrogen) to plates and confirmed by immunoblot using an anti-Flag antibody (Sigma-Aldrich).

### Construction of *icaA* ^*-*^ mutants of *C. burnetii*

A PCR product consisting of CBU1823 and 2 kb of DNA flanking either end was amplified from *C. burnetii* (Nine Mile RSA 493 phase II) genomic DNA by using PfuUltra II Fusion HotStart DNA polymerase (Agilent Technologies). The product was ligated into the *Bam*HI and *Sal*I sites of pJC-CAT. The resulting construct, pJC-1823FL, was then used as a template in a PCR. The resulting PCR product was then gel purified and digested with *Not*I-HF (New England Biolabs) and ligated with the kanamycin resistance gene amplified from pJB-Kan. The ligation mix was then transformed into DH5α. The resulting construct, pJC1823KO, in which the CBU1823 gene was replaced with a kanamycin resistance gene, was then used in the gene knockout experiment^66^. CBU1823 knockout was confirmed by Southern hybridization using probes directly binding to CBU1823 and the kanamycin resistance cassette.

### Bone marrow-derived and alveolar macrophages

AMs were obtained by bronchoalveolar lavage with warm PBS containing 5 mM EDTA. AMs were washed twice with PBS, seeded on tissue culture plates with RPMI 1640 media 10% FBS, 2 mM L-glutamine and cultivated at 36 °C, 5% CO_2_. *C. burnetii* replication in AMs was assessed by quantitative PCR, as described previously^67^. Bone marrow cells were collected from femurs and differentiated with RPMI 1640 (Sigma-Aldrich), 20% FBS (Invitrogen), 30% L-929 cell-conditioned media (LCCM), 2 mM L-glutamine (Sigma-Aldrich) and 100 U ml^−1^ penicillin–streptomycin (Sigma-Aldrich) cultivated at 36 °C, 5% CO_2_. BMDMs were collected and seeded on tissue culture plates and kept in RPMI 1640 media 10% FBS, 5% LCCM and 2 mM L-glutamine^68^.

### Construction of retroviral expression vectors and transduction of BMDMs

Full-length genomic C57BL/6 *Casp11* was amplified by PCR with sequence-specific primers containing *Not*I and *Xho*I sites for cloning into pCDNA3.1/Hygro^+^ vector (Invitrogen). *Casp11* was subsequently subcloned into a pMSCV2.0 murine-specific retroviral vector after the digestion of the purified pCDNA3 construct with *Not*I and *Sal*I. pCL vector system^69^ for packaging retroviruses was co-transfected with pMSCV–Casp11–IRES–GFP using polyethylenimine in monolayers of Peak cells (ATCC) maintained in RPMI 1640 with 10% FBS at 36 °C and 5% CO_2_. BMDMs in day 3 of differentiation were collected, spun down and resuspended in retrovirus-containing peak cell supernatant supplemented with RPMI 1640 containing 20% FBS and 25% LCCM. Cell transduction was confirmed by fluorescence microscopy. BMDMs were then collected, seeded on tissue culture plates one day before infection and kept in RPMI 1640 media 10% FBS and 5% LCCM.

### Infection conditions

*C. burnetii* from frozen stocks was thawed at 37 °C. *L. pneumophila* single colonies were seeded and grown for 2 days in CYE agar plates, collected, diluted in distilled water and quantified by measuring optical density (OD) at 600 nm. *L. pneumophila* mutants expressing *C. burnetii* effectors were infected in the presence of 1 mM IPTG. *E. coli* single colonies were grown in LB broth for 8 h and quantified by measuring OD at 600 nm. *E. coli-*infected macrophages were washed twice with warm PBS one hour after infection, and the media were replaced with media containing gentamicin (Sigma-Aldrich, 50 μg ml^−1^). When necessary, the plates were centrifuged for 15 min at 300 *g* to ensure the comparable adhesion of the bacteria to the cells.

### Extraction of *C. burnetii* LPS

LPS was extracted using a modified hot phenol method, as described previously^70^. Briefly, 10^6^ *C. burnetii* were suspended in 1 ml of 50% phenol (pH 8.8), boiled for 10 min, incubated for 5 min on ice and then centrifuged at 14,000 *g* for 5 min. The aqueous phase was collected, and the extraction was repeated on the pellet. The aqueous phases from both extractions were pooled and vacuum dried for 17 h. The pellet was dissolved in 100 μl of ultra-pure distilled H_2_O. The LPS content after extraction was estimated by a phenol–sulfuric acid method of carbohydrate quantification. Approximately, 1 μg ml^−1^ of *C. burnetii* LPS were used to transfect BMDMs using DOTAP (Roche) according to the manufacturer's instructions.

### Determination of *L. pneumophila* number per *Legionella*-containing vacuole (LCV)

BMDMs were cultivated in coverslips and fixed with 4% PFA after infection. The coverslips were immunostained with rabbit anti-*Legionella* (1:2,000) and goat anti-rabbit IgG Alexa Fluor 488 (Molecular Probes), mounted in glass slides with ProLong Gold containing DAPI (Molecular Probes). The coverslips were analysed with a fluorescence microscope (Leica, Germany), and the number of bacteria per LCV was estimated visually.

### Immunoblot analysis

BMDMs were lysed with a RIPA buffer (10 mM Tris-HCl (pH 7.4), 1 mM EDTA, 150 mM NaCl, 1% Nonidet P-40, 1% (w/v) sodium deoxycholate and 0.1% (w/v) SDS) supplemented with a protease inhibitor cocktail. Precleared lysates and supernatants were boiled with Laemmli buffer, resolved by SDS–polyacrylamide gel electrophoresis and transferred to a 0.22-μm nitrocellulose membrane (GE Healthcare). Rat anti-caspase-1 p20 monoclonal antibody clone 4B4 (Genentech, 1:500), mouse anti-caspase-1 p10 (Santa Cruz Biotechnology, 1:250), goat anti-IL-1β p17 subunit (Sigma-Aldrich, 1:250), rat anti-caspase-11 p10 (Abcam, 1:500) and rabbit anti-beta actin (Sigma-Aldrich, 1:5,000) were used for antigen detection. To detect the expression of Flag-fused effectors by *L. pneumophila*, bacterial heavy patches streaked from CYE plates after 2 days of growth in the presence of 1 mM IPTG were boiled in Laemmli buffer, immunoblotting was carried out as described above, and anti-Flag M2 (Sigma-Aldrich, 1:5,000) antibody was used for antigen detection.

### Reagents for cell stimulation and cytokine determination

Ultra-pure LPS (*E. coli* K12) and poli I:C were from Invivogen. Tumour-necrosis factor-α was from eBioscience. ATP, nigericin and CTB were from Sigma-Aldrich. Cytokines from supernatants of the BMDM culture were detected by ELISA (OptEIA, BD Biosciences), according to the manufacturer's instructions.

### Membrane pore formation assay

Pore formation in BMDMs and AMs was quantified by the permeability to ethidium bromide in damaged cells^57^. Images were acquired with a fluorescence microscope (Leica, Germany), five fields per coverslip. Pore formation was quantified using ImageJ software (NIH). For the kinetics of pore formation, the uptake of PI in infected cells was evaluated^13^. BMDMs were seeded in a black, clear-bottom 96-well plate. Before infection or stimulation, BMDMs were washed with warm PBS, and the media were replenished with RPMI 10% without phenol red, 0.038 g ml^−1^ NaHCO_3_, 6 μg ml^−1^ PI. Infected BMDMs were kept at 37 °C, and PI was excited at 538 nm. The fluorescence emission was read at 617 nm at every 5 min using a plate fluorometer (FlexStation 3, Molecular Devices).

### FLICA staining and flow cytometry analysis

BMDMs were stained with a caspase-1 FLICA probe (FAM-YVAD-FMK), as recommended by the manufacturer. HEK293 reporter cells were likewise stained with a pan-caspase FLICA probe (carboxyfluorescein-VAD-fluoromethylketone, FAM-VAD-FMK; ImmunoChemistry Technologies). Data from 30,000 events were acquired on a FACSCanto II (BD Biosciences) and were analysed with FlowJo software (Tree Star).

### Active caspase-11 pull-down assay

The media of primed BMDMs were replenished with fresh media containing 20 μM biotin-VAD-FMK (Enzo) 15 min before infection. Infected BMDMs were lysed in RIPA buffer (10 mM Tris-HCl (pH 7.4), 1 mM EDTA, 150 mM NaCl, 1% Nonidet P-40, 1% (w/v) sodium deoxycholate and 0.1% (w/v) SDS) supplemented with a protease inhibitor cocktail (Roche). Cleared lysates were equalized according to total protein content, incubated overnight with streptavidin–agarose beads (Novex) and thoroughly rinsed with RIPA buffer. Bound proteins were eluted by re-suspension in Laemmli sample buffer, boiled for 5 min and separated by SDS–polyacrylamide gel electrophoresis.

### Statistical analysis

The data were plotted and analysed using GraphPad Prism 5.0 software. The statistical significance was calculated using Student's *t-*test or analysis of variance (ANOVA). Differences were considered statistically significant when the *P* value was <0.05.

## Additional information

**How to cite this article:** Cunha, L. D. *et al.* Inhibition of inflammasome activation by *Coxiella burnetii* type IV secretion system effector IcaA. *Nat. Commun.* 6:10205 doi: 10.1038/ncomms10205 (2015).

## Supplementary Material

Supplementary Information Supplementary Figures 1-10 and Supplementary Table 1
