# Supplementary material for: Inhibition of inflammasome activation by Coxiella burnetii type IV secretion system effector IcaA
Source: Nat Commun. 2015 Dec 21;6:10205. doi: 10.1038/ncomms10205 (PMC4703858; doi:10.1038/ncomms10205)
Supplement: Supplementary Information — Supplementary Figures 1-10 and Supplementary Table 1 [file ncomms10205-s1.pdf]

Supplementary Information

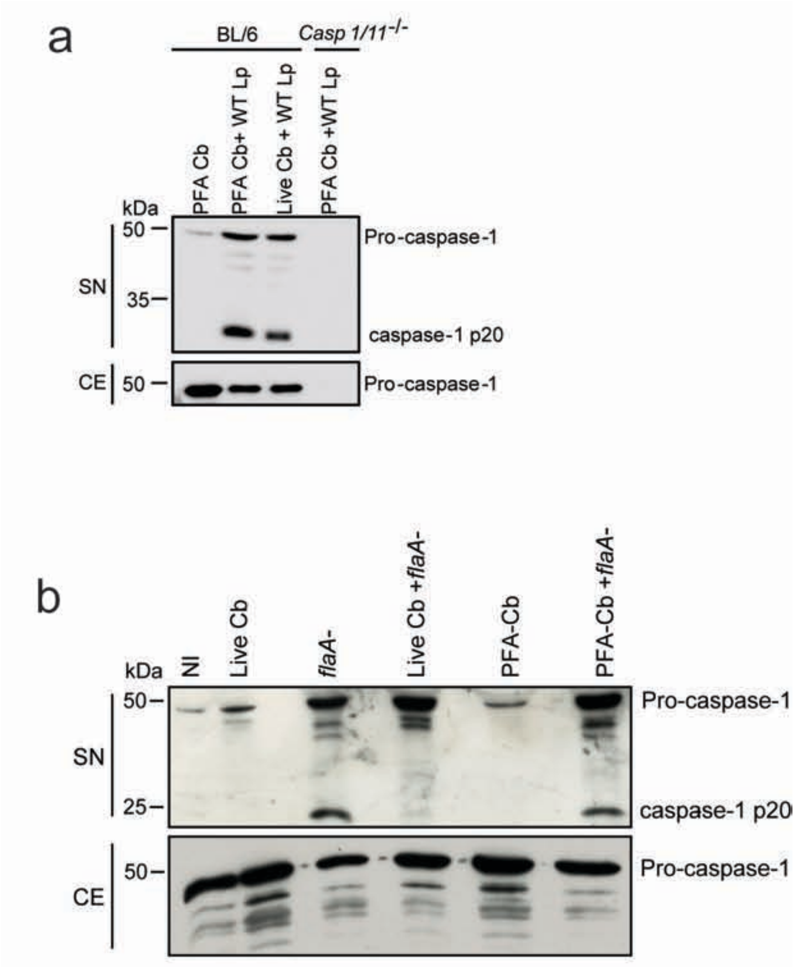

Supplementary Figure 1. Viable *C. burnetii* is required for inhibition caspase-1 activation induced by *L. pneumophila*. Bone marrow-derived macrophages (BMDM) were left uninfected (NI) or were infected with PFA-fixed (PFA Cb) or live *C. burnetii* (Live Cb) at MOI 30 for 24 h and further infected with wild-type *L. pneumophila* (WT Lp, MOI 10) or *flaA*$^-$ mutant (*flaA*-) for 9 h. Co-infected BMDM are indicated (Cb + WT Lp or Cb + *flaA*$^-$). (**a**) Immunoblot showing levels of processed p20 subunit of caspase-1 (caspase-1 p20) and unprocessed caspase-1 (pro-caspase-1), as determined in supernatant (SN) and cell extract (CE). (**b**) Similar in (**a**) for BL/6 BMDM.

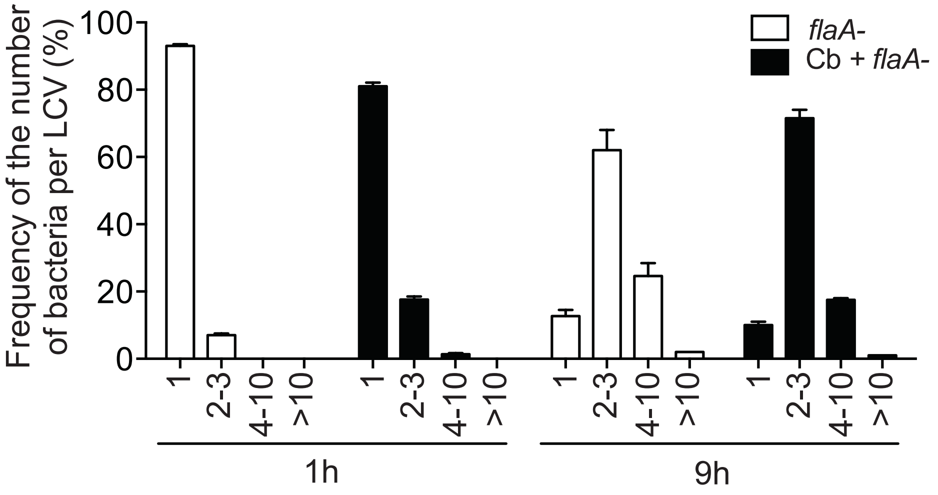

Supplementary Figure 2. Co-infection with *C. burnetii* does not interfere with *flaA- L. pneumophila* replication in BMDM. Bone marrow-derived macrophages (BMDM) from C57BL/6 mice were left uninfected or were infected with *C. burnetii* at MOI 30 for 24 h and further infected with *flaA*$^-$ *L. pneumophila* strain Lp01 (*flaA-*, MOI 10) for 9 h. Co-infected BMDM are indicated (Cb + *flaA-*). An estimation of the frequency of bacteria per *Legionella*-containing vacuole (LCV), as assessed by counting on the fluorescence microscopy, is shown.

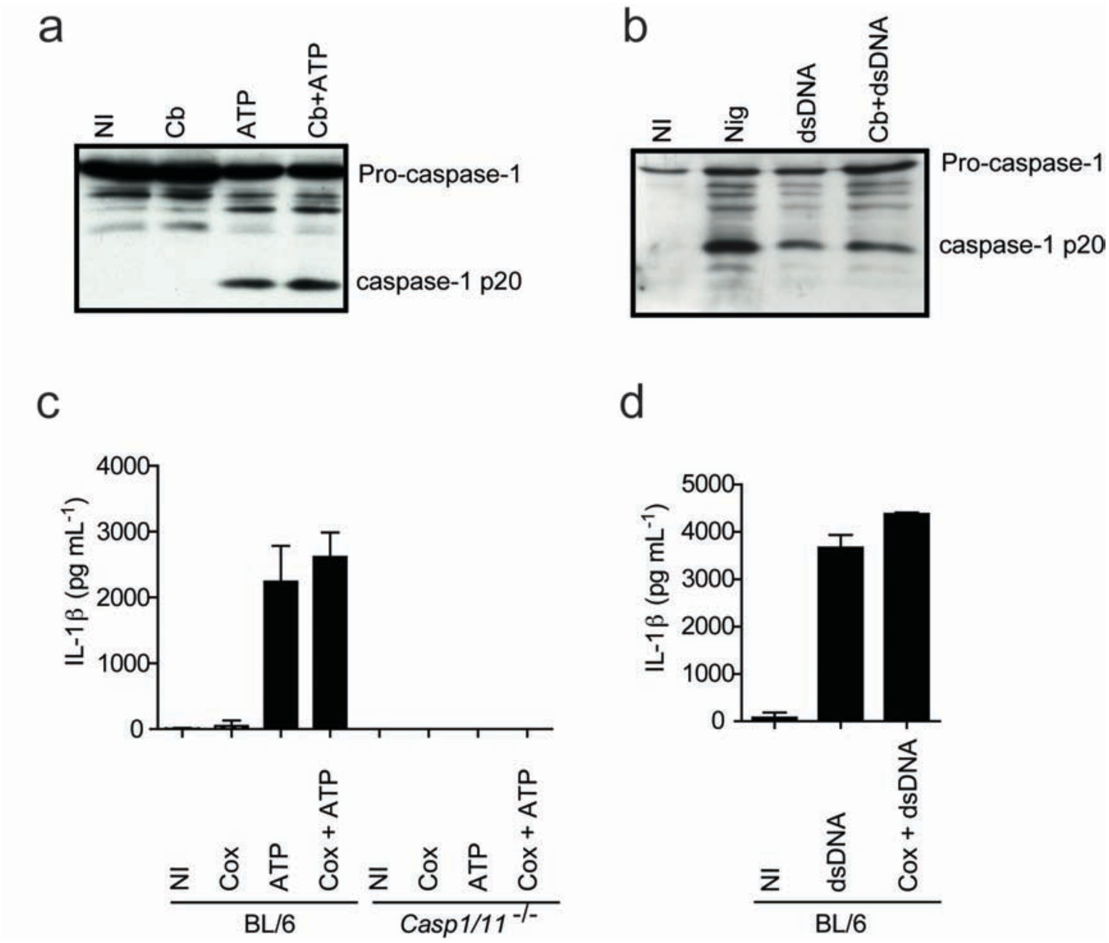

Supplementary Figure 3. ***C. burnetii*** **does not interfere with ATP-mediated activation of the canonical NLRP3 inflammasome or with dsDNA-mediated activation of the AIM2 inflammasome.** Bone marrow-derived macrophages (BMDM) were left uninfected (NI) or were infected with *C. burnetii* (Cb, MOI 30) for 24 h and further stimulated with LPS (1 μg ml^{-1}) for 4 h. The cells were then treated with either ATP (5 mM) for 20 min or nigericin (20 mM) for 40 min or were transfected with 1 μg of dsDNA for 6 h. (**a, b**) Immunoblot showing levels of processed p20 subunit of caspase-1 (caspase-1 p20) and unprocessed caspase-1 (pro-caspase-1), as determined in supernatant (SN) and cell extract (CE) of BL/6 BMDM. (**c, d**) IL-1β secretion in the supernatant was estimated by ELISA.

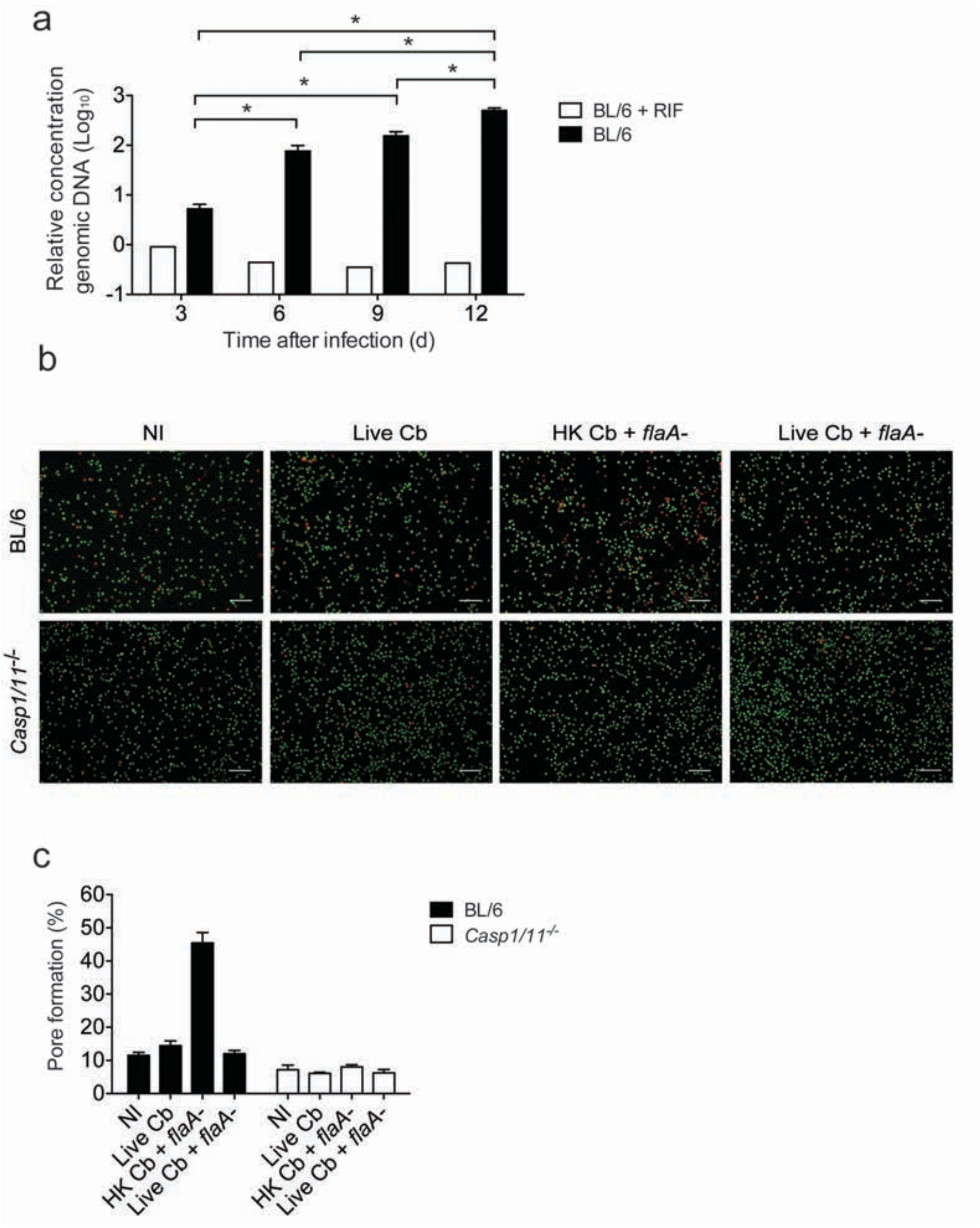

Supplementary Figure 4. *C. burnetii* inhibits pore formation in response to *flaA- L. pneumophila* in alveolar macrophages. (**a**) Alveolar macrophages (AM) from C57BL/6 mice were infected with *C. burnetii* (MOI 3), and bacterial growth was assessed by qPCR. Relative bacterial replication was calculated as $\log(Y_{x=n}) - \log(Y_{x=0})$. Control AM were treated with bacterial translation inhibitor rifampicin (RIF, 20 mg mL^{-1}). Data are expressed as the average ± SEM of triplicate wells and significance was calculated with ANOVA. *, $P<0.05$. (**b,c**) AM were left uninfected (NI) or were infected with heat-killed (HK Cb) or live *C. burnetii* (Live Cb) at MOI 30 for 6 h and further infected with *flaA$^-$ L. pneumophila* (*flaA*-, MOI 10) for 3 h. (**b**) Fluorescence micrographs revealing cell permeability, as assessed by the quantification of EtBr influx (red dots) in acridine orange (green) stained AM. Scale bar corresponds to 75 μm. (**c**) Quantification of the experiment shown in (**b**).

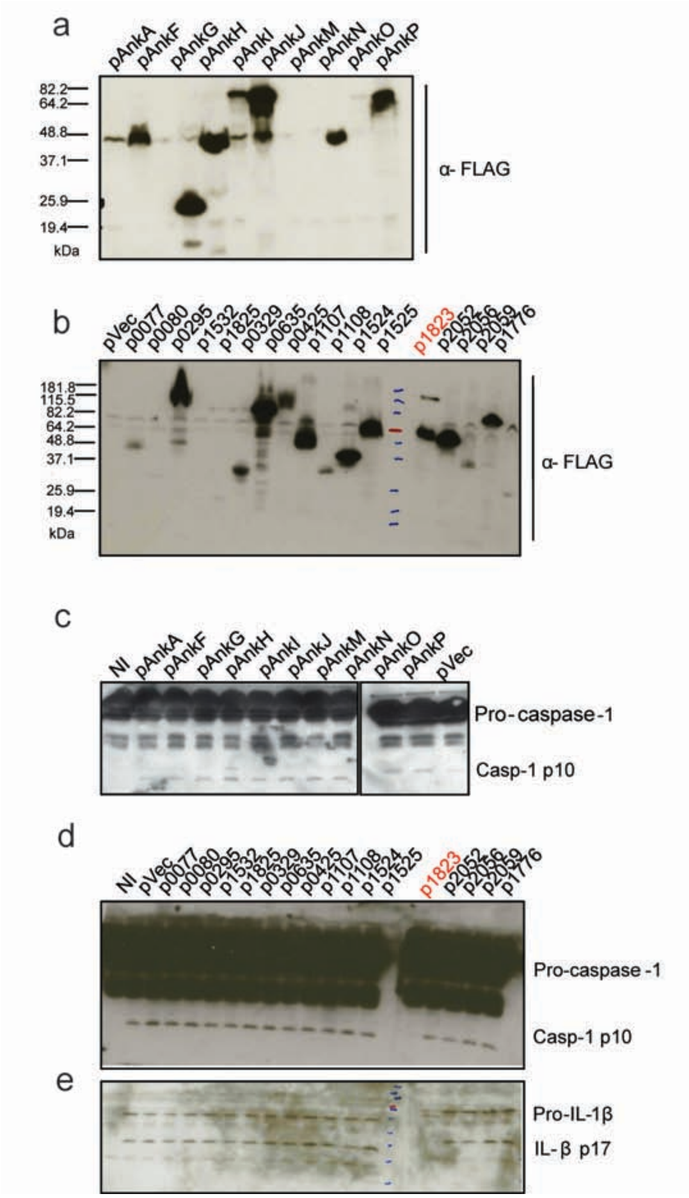

Supplementary Figure 5. Identification of CBU1823 (IcaA) as an effector protein that inhibits a flagellin-independent pathway for caspase-1 activation induced by *L. pneumophila*. Bone marrow-derived macrophages (BMDM) from C57BL/6 mice were infected with *flaA*⁻ *L. pneumophila* expressing each indicated effector (indicated is the CBU number or the protein name) or an empty vector (pVec) for 9 h in the presence of 1 mM IPTG. (**a, b**) Mutants were grown in CYE agar plates for two days, and effector expression was assessed by immunoblot for the Flag epitope tag (expression of N-terminal Flag fusions). (**c, d**) Immunoblot showing levels of processed p10 subunit of caspase-1 (caspase-1 p10) and unprocessed caspase-1 (pro-caspase-1) in supernatant. (**e**) Immunoblot showing levels of p17 subunit of mature IL-1β (IL-1β p17) and pro-IL-1β in supernatant fraction. CBU1823 (IcaA) is highlighted in red.

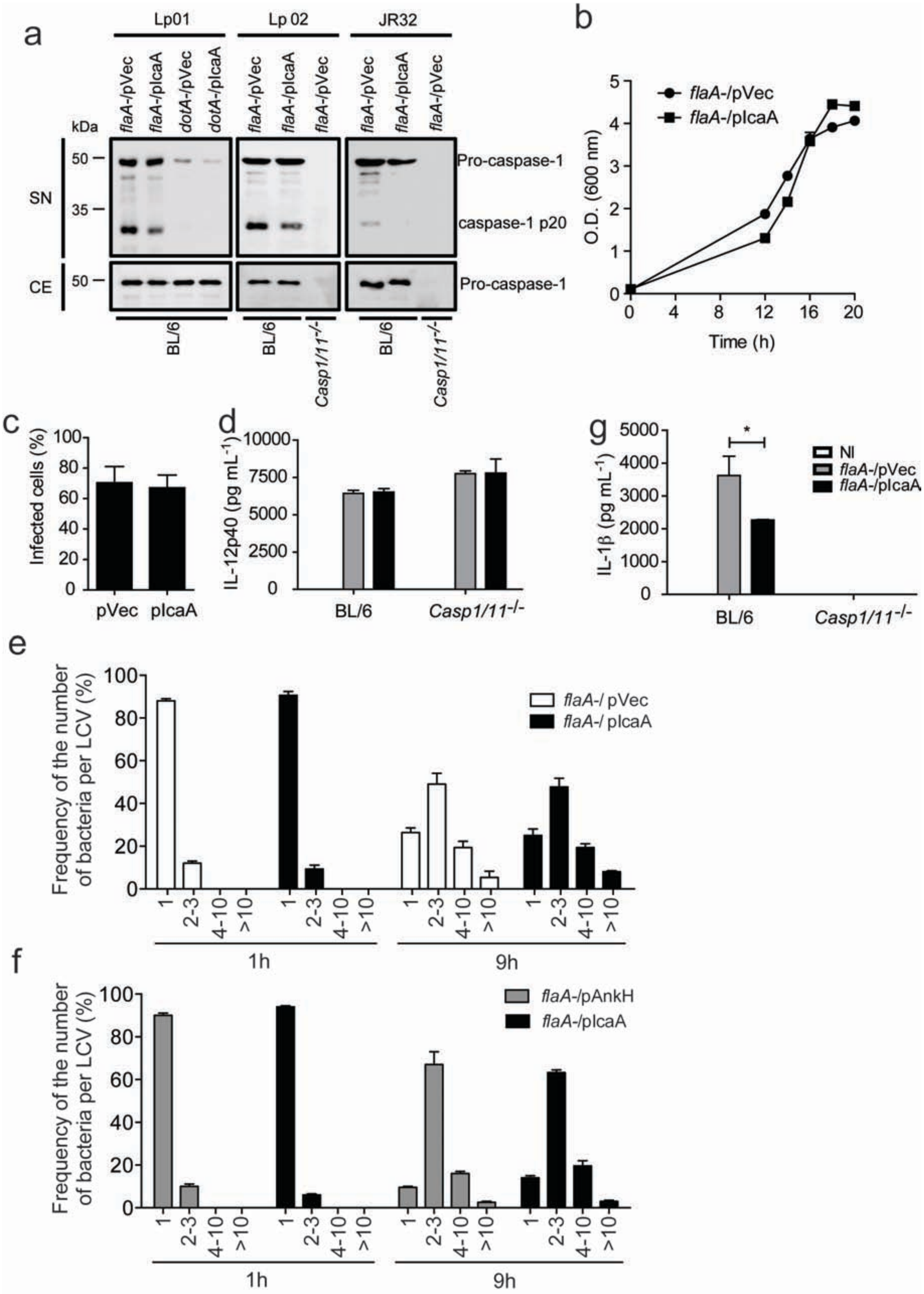

a
Lp01
Lp 02
JR32
flaA-/pVec
flaA-/pIcaA
dotA-/pVec
dotA-/pIcaA
kDa
50
35
SN
CE
Pro-caspase-1
caspase-1 p20
Pro-caspase-1
BL/6
Casp1/11-/-
b
flaA-/pVec
flaA-/pIcaA
O.D. (600 nm)
Time (h)
c
Infected cells (%)
pVec
pIcaA
d
IL-12p40 (pg mL-1)
BL/6
Casp1/11-/-
g
IL-1β (pg mL-1)
*
NI
flaA-/pVec
flaA-/pIcaA
e
Frequency of the number of bacteria per LCV (%)
flaA-/ pVec
flaA-/ pIcaA
1
2-3
4-10
>10
1h
9h
f
Frequency of the number of bacteria per LCV (%)
flaA-/pAnkH
flaA-/pIcaA

Supplementary Figure 6. Expression of IcaA by *L. pneumophila* interferes with caspase-1 activation but does not influence the replication in axenic media, macrophage infection or the induction of IL-12p40. *flaA*$^-$ or *dotA*$^-$ mutants from three different *L. pneumophila* strains: Lp01, thymidine auxotroph Lp02 and JR32, transformed with the empty vector (pVec), pSN85 encoding IcaA (pIcaA) or pSN85 encoding AnkH (pAnkH) were evaluated. (**a**) Immunoblot showing levels of processed p20 subunit of caspase-1 (caspase-1 p20) and unprocessed caspase-1 (pro-Casp-1), as determined in supernatant (SN) and cell extract (CE) of bone marrow-derived macrophages (BMDM) infected for 9 h. (**b**) Replication of *flaA*$^-$/pVec and *flaA*$^-$/pIcaA (strain Lp01) in AYE broth was assessed by optic density (OD) at 600 nm. (**c**) BL/6 BMDM were infected with *flaA*$^-$/pVec and *flaA*$^-$/pIcaA (strain Lp01) for 1 h, and bacterial internalization was assessed by fluorescence microscopy using anti-*L. pneumophila*. (**d**) IL-12p40 secretion in the supernatant of BMDM infected for 9 h with *flaA*$^-$/pVec and *flaA*$^-$/pIcaA (strain Lp02), as estimated by ELISA. (**e, f**) Estimation of the frequency of bacteria per *Legionella*-containing vacuole (LCV) of BMDM infected with *flaA*$^-$/pVec and *flaA*$^-$/pIcaA and *flaA*$^-$/pAnkH (strain Lp01), as assessed by counting on fluorescence microscopy. (**g**) IL-1β secretion in the supernatant of BMDM infected for 9 h with *flaA*$^-$/pVec and *flaA*$^-$/pIcaA (strain Lp02), as estimated by ELISA. Data are expressed as the average ± SEM of triplicate wells and significance was calculated with ANOVA. *, $P<0.05$.

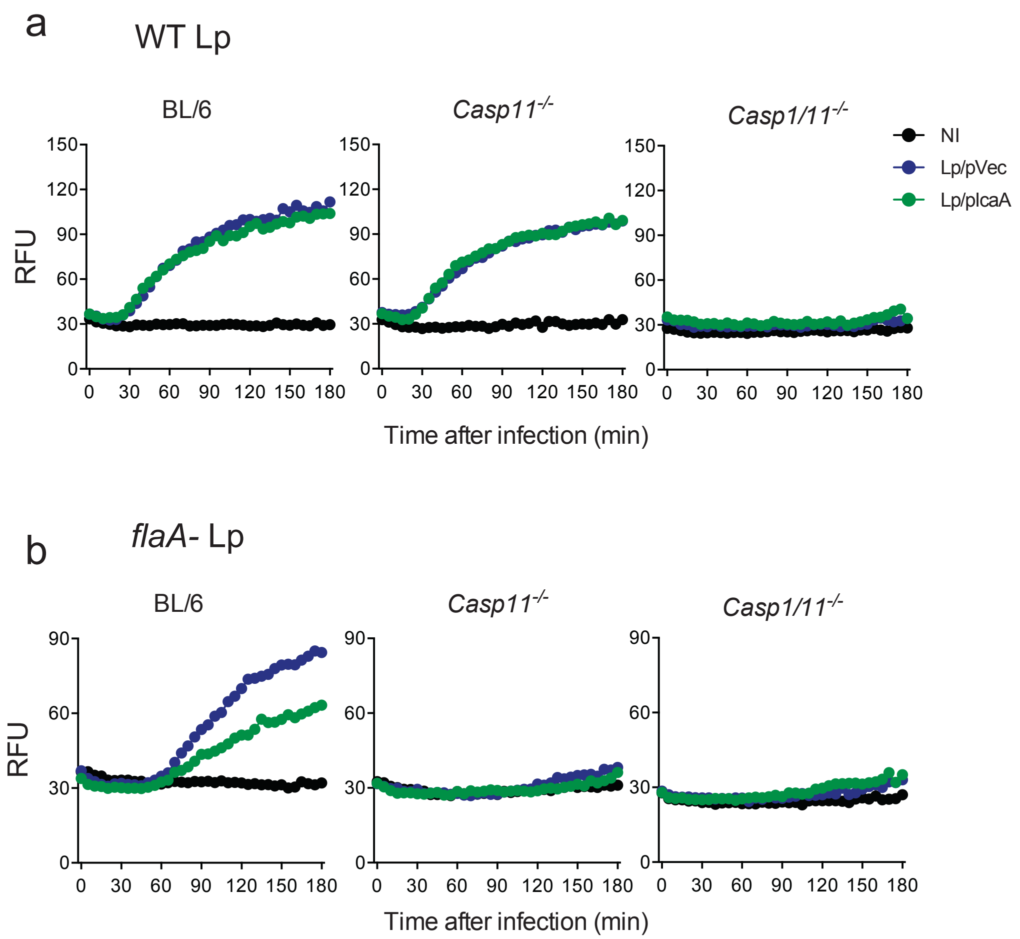

Supplementary Figure 7. IcaA inhibits caspase-11-dependent pore formation in response to *flaA*- *L. pneumophila*. Bone marrow-derived macrophages (BMDM) from C57BL/6 or isogenic mice deficient in caspase-11 or caspase-1/11 were primed with LPS (0.5 mg mL^{-1}) for 3 h. Cells were left uninfected (NI) or were infected with wild-type (**a**) or *flaA*- *L. pneumophila* (**b**) transformed with either an empty vector (Lp/pVec) or a vector encoding IcaA (Lp/pIcaA) at MOI 10. Pore formation was assessed fluorometrically in real time by the uptake of propidium iodide (RFUs, relative fluorescence units).

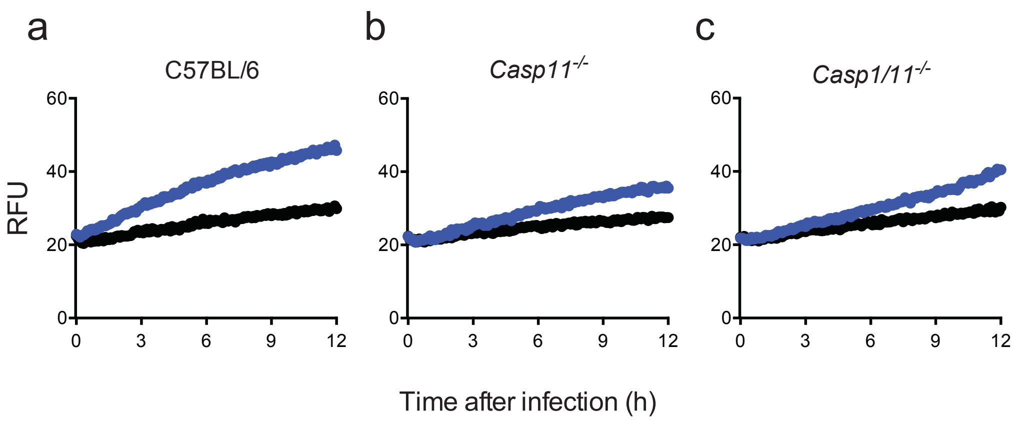

Supplementary Figure 8. *C. burnetii* LPS induces pore formation when transfected in macrophages. Bone marrow-derived macrophages (BMDM) generated from C57BL/6 (**a**), *Casp11*$^{-/-}$ (**b**) or *Casp1/11*$^{-/-}$ (**c**) mice were primed with poly I:C (5 μg ml^{-1}) for 4 h. Cells were transfected with 1 μg ml^{-1} *C. burnetii* LPS (blue) or with MOCK (black) for up to 12 hrs. Pore formation was assessed fluorometrically in real time by the uptake of propidium iodide (RFUs, relative fluorescence units).

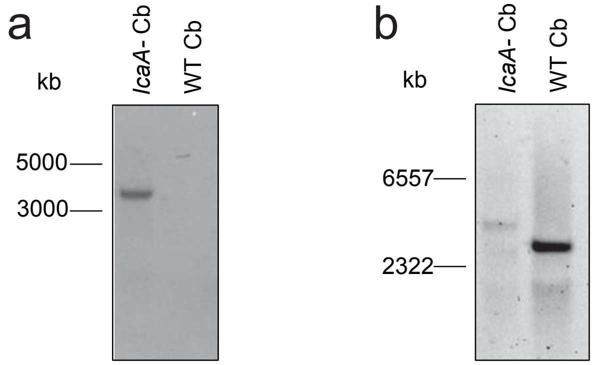

Supplementary Figure 9. Validation of the *icaA* mutant *C. burnetii.* CBU_1823 (*icaA*$^{-}$) knockout was confirmed by southern hybridization using probes to *icaA* and the kanamycin resistance cassette. (**a**) Southern blot with probe against kanamycin resistance cassette. (**b**) Southern blot with probe against *icaA.*

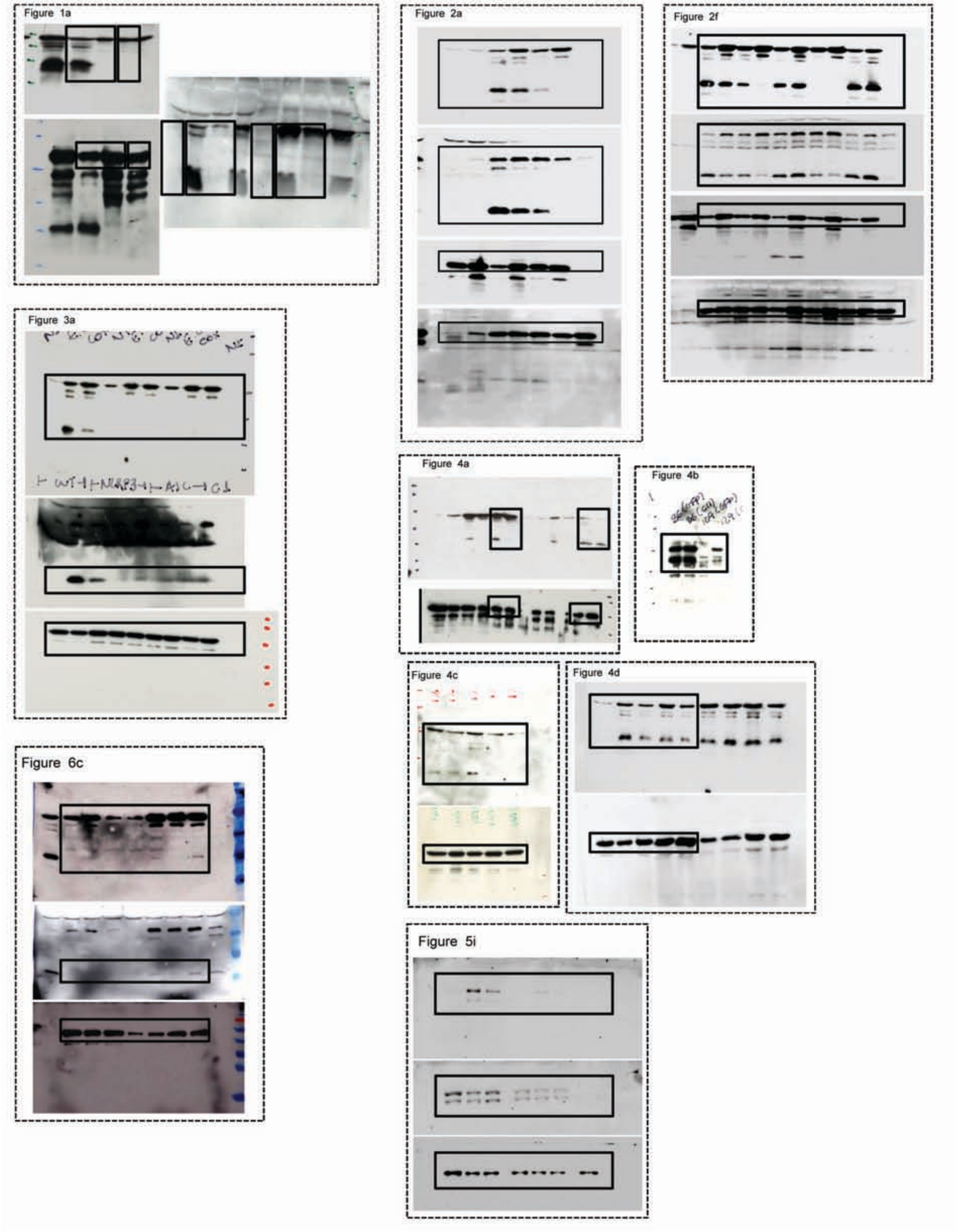

Supplementary Figure 10. Full immunoblots of main figures presented in this study.

Suplemmentary Table1. List of the primers used in this study .

Primer	Sequence 5' to 3'	Purpose
AnkA F	AAggatccTCTTGCTTAGCTTAATGG	pSN85
AnkA R	AAgcatgcTTAAAACAGTCCGGGGCC	pSN85
AnkB F	AAtctagaGTATGTTTAACCAATTGGA	pSN85
AnkB R	GGgcatgcTTACATGTGCTTACCCGG	pSN85
AnkF F	AAggatccTCATGAGACAGCGTGAAA	pSN85
AnkF R	AAgcatgcCTACCGCTGGAAGCCGC	pSN85
AnkG F	AAggatccTCATGAGTAGACGTGAGA	pSN85
AnkG R	AAgcatgcTCACCGAGGACTAGACAG	pSN85
AnkH F	AAggatccTCTTGTCAGAATTAGGAG	pSN85
AnkH R	AAgcatgcTTAGATTAACGTGCGCGT	pSN85
AnkI F	AAggatccTCATGAGAGAATCATCAG	pSN85
AnkI R	AAgcatgcCTAAATTCCAAAAGAACC	pSN85
AnkJ F	AAggatccTCATGGCGAAATTTACTA	pSN85
AnkJ R	AAgcatgcTTACGCAGCGCGCATGGT	pSN85
AnkN F	AAggatccTCTTGCGAGAGCTTATGA	pSN85
AnkN R	AAgcatgcCTATAGACCGTATTGCTG	pSN85
AnkO F	AAggatccTCATGGAAATAATTTCCTT	pSN85
AnkO R	AAgcatgcTTAATAACGATTTTTTGTT	pSN85
AnkP F	AAggatccTCGTGGGACAAAATACAA	pSN85
AnkP R	AAgcatgcCTTAAGCTAAGCAAGGGG	pSN85
CBU1823_1 FOR	GACggatccATTCCTAAGGTGGATATGTGGGC	pJC1823-FL
CBU1823_1 REV	GACgtcgacCGAGTTGGTTTTTCATTGCCAAGG	pJC1823-FL
CBU1823_2 FOR	GATgtcgacgcggccgcgcatgcATTTCCCTCCGCAATAGCTTA	pJC1823-KO
CBU1823_2 REV	GATgcatgcgcggccgcCTTGCCTTTTACCCCCTCGT	pJC1823-KO
Kan FOR	GACgcggccgcAGCTTATGGCTTCGTTTCGCAG	pJC1823-KO
Kan REV	GACgcggccgcTCAGAAGAACTCGTCAAGAAGGCG	pJC1823-KO
CBU1823_SH F	CCATTCAAGAGAGTTCATGTATGCCATCA	Southern hybridization
CBU1823_SH R	ATCTCATCTCCTTCAGCCTGACTT	Southern hybridization
Kan_SH F	GTGGAGAGGCTATTCGGCTATG	Southern hybridization
Kan_SH R	TTGAGCCTGGCGAACAGTT	Southern hybridization
Cb dotA F1	GCGCAATACGCTCAATCACA	qPCR
Cb dotA R1	CCATGGCCCCAATTCTCTT	qPCR
